# Interleukin-10 genetically modified clinical-grade mesenchymal stromal cells markedly reinforced functional recovery after spinal cord injury via directing alternative activation of macrophages

**DOI:** 10.1186/s11658-022-00325-9

**Published:** 2022-03-17

**Authors:** Tianyun Gao, Feifei Huang, Wenqing Wang, Yuanyuan Xie, Bin Wang

**Affiliations:** Center for Clinic Stem Cell Research, the Affiliated Drum Tower Hospital of Nanjing University Medical School, Nanjing, 210008 Jiangsu China

**Keywords:** MSCs, Interleukin 10, Gene modification, Spinal cord injury, Cell-based therapy, Cell quality assessment

## Abstract

**Background:**

After spinal cord injury (SCI), dysregulated or nonresolving inflammatory processes can severely disturb neuronal homeostasis and drive neurodegeneration. Although mesenchymal stromal cell (MSC)-based therapies have showed certain therapeutic efficacy, no MSC therapy has reached its full clinical goal. In this study, we examine interleukin-10 (IL10) genetically modified clinical-grade MSCs (IL10-MSCs) and evaluate their clinical safety, effectiveness, and therapeutic mechanism in a completely transected SCI mouse model.

**Methods:**

We established stable IL10-overexpressing human umbilical-cord-derived MSCs through electric transduction and screened out clinical-grade IL10-MSCs according to the criteria of cell-based therapeutic products, which were applied to mice with completely transected SCI by repeated tail intravenous injections. Then we comprehensively investigated the motor function, histological structure, and nerve regeneration in SCI mice, and further explored the potential therapeutic mechanism after IL10-MSC treatment.

**Results:**

IL10-MSC treatment markedly reinforced locomotor improvement, accompanied with decreased lesion volume, regeneration of axons, and preservation of neurons, compared with naïve unmodified MSCs. Further, IL10-MSC transplantation increased the ratio of microglia to infiltrated alternatively activated macrophages (M2), and reduced the ratio of classically activated macrophages (M1) at the injured spinal cord, meanwhile increasing the percentage of Treg and Th2 cells, and reducing the percentage of Th1 cells in the peripheral circulatory system. In addition, IL10-MSC administration could prevent apoptosis and promote neuron differentiation of neural stem cells (NSCs) under inflammatory conditions in vitro.

**Conclusions:**

IL10-MSCs exhibited a reliable safety profile and demonstrated promising therapeutic efficacy in SCI compared with naïve MSCs, providing solid support for future clinical application of genetically engineered MSCs.

**Supplementary Information:**

The online version contains supplementary material available at 10.1186/s11658-022-00325-9.

## Introduction

In mammals, the spinal cord connects most of the peripheral nervous system to the brain and is protected by a vertebral column divided into multiple segments. Different segments of spinal nerves innervate different regions of the body [1, 2]. In addition to acting as a conduit for the transmission of sensory input to the brain and motor output to effector tissues, the spinal cord is also responsible for generating spinal cord reflexes and protecting the body from harmful stimuli [3]. Various types of spinal cord injury (SCI) could cause the loss of nerve connections and severe damage of autonomic functions, resulting in permanent motor disorders or even complete paralysis [4]. To date, there is no effective therapy for SCI in clinic [5]. For example, trauma injury can lead to lower motor neuron (LMN) apoptosis, and the axon is separated from the neuromuscular junction (NMJ), leaving a denervated motor endplate [6]. After SCI, the blood–spinal cord barrier is damaged, a variety of immune cells infiltrate into the local microenvironment at the lesion site, and peripheral blood mononuclear cells also immediately infiltrate and differentiate into macrophages of two phenotypes based on molecular phenotype and effector function: classically activated pro-inflammatory (M1) and alternatively activated anti-inflammatory (M2) cells [7], which play a pro-inflammatory and anti-inflammatory role during the progression and recovery of SCI, respectively.

Microglia cells are native immune cells in the central nervous system (CNS) [8–10]. Microglia and infiltrating macrophages both play two distinct roles in regulating immune and inflammatory responses after SCI [11]. During SCI, activated microglia/macrophages can promote nerve repair by increasing revascularization and axon regrowth. However, they can also cause pathological reactions in injured tissue through releasing reactive oxygen species (ROS), neurotoxins, and pro-inflammatory cytokines, and even lead to axon retraction and death [12]. Classical M1-type polarized microglia/macrophages release destructive pro-inflammatory mediators, aggravating tissue damage. In contrast, immune cells with an alternative anti-inflammatory M2 phenotypes help resolve local inflammation, remove cell debris, and provide nutritional factors [13, 14]. Therefore, facilitating M2 polarization of microglia/macrophages from M1 may be necessary to tissue preservation and repair after SCI. In fact, after SCI, M1 microglia/macrophages are rapidly induced and concentrated near the epicenter. In the acute phase of SCI, the number of M2 microglia/macrophages only temporarily increases, which is much lower than that of M1 phenotypes [15]. Therefore, increasing and prolonging the M2 macrophage subtype in the injured local microenvironment may represent a promising strategy for tissue repair after SCI.

Interleukin-10 (IL10) is an anti-inflammatory cytokine that helps to regulate the immune response and induces macrophages to polarize toward anti-inflammatory phenotypes [16]. Owing to the direct inhibition of the pro-inflammatory cytokine [17], IL10 can also reduce the proliferation of astrocytes after brain trauma [7]. Neuroprotective effects of IL10 have been reported in several rodent SCI models, including directly providing nutritional support to neurons [18], reducing inflammation through binding with IL10 receptors [19], and increasing formation of myelination [20]. However, as IL10 protein has a short half-life, only about 1–2 min in vivo, it is difficult to sustain an effective dose when applied clinically.

Mesenchymal stromal cells (MSCs), especially human umbilical-cord-derived MSCs (hUCMSCs), have a variety of advantages, including easy expansion and without ethical issues as a type of practical seeding cell for tissue regeneration [21, 22]. The hUCMSCs also are easy to modify genetically and very suitable as carriers for delivering key target genes [23]. Studies have found that transplantation of hUCMSCs can inhibit the formation of glial scars after SCI, promote nerve regeneration, and significantly improve motor nerve function [24]. Therefore, MSC-based therapy is a very promising strategy for SCI. However, many clinical trials based on stem cell therapies have failed to deliver desired results. There are several problems hindering the translation of cell-based therapy in clinic. One is that the same type of cell is used to treat many different diseases caused by different etiologies, without establishing specific standards for cell quality based on the characteristics of each disease. Second, the therapeutic activity of the naïve seeding cells is limited, insufficient to reach the desired therapeutic effects. Therefore, it is necessary to develop novel stem-cell-based therapy strategies such as establishing specific quality criteria of seeding cells for corresponding disease, increasing biological activity by preconditioning, and reinforcing therapeutic efficacy by specific gene modification. The seeding cells with genetic modification have more advantages, including efficient and long-term expression of effector molecules and/or targeted molecules to exert higher therapeutic effects. However, gene modification for cell-based product will face the challenge of ensuring the genetically modified cells meet the quality criteria of a cell-based therapeutic product, to avoid potential risk to recipients when used in clinic. Although there have been many studies regarding gene modification in cell-based therapy, they lack systematic quality assessments for a cell-based therapeutic product. For clinical application, it is essential to develop a standardized protocol to guarantee the safety and efficacy of gene-modified cell therapeutic products [25].

Another unanswered issue in the clinical application of MSCs is their optimal transplantation route and strategy. Previous studies have confirmed that MSCs can be delivered to the injured spinal cord via several routes, including intraoperative injection [26], lumbar puncture [27], and intravenous injection [28, 29]. In clinic, the least invasive cell delivery strategy is preferred. Clinically, repeated injections of seeding cells directly into the injured spinal cord are not allowed owing to the possible damage caused by local injections. It is well known that MSCs have a tropism to the injury site [30], which supports the use of repeated systemic injection to obtain better therapeutic effects. Therefore, intravenous injection is a better choice for clinical use because it is safer and allows multiple administrations. This strategy has been successfully tested in several experimental studies [31–33].

Our team has established a center for clinical-grade stem cell preparation and quality inspection, fully compliant with current “Good Manufacturing Practice” (GMP) guidelines [20]. In this study, we introduced human *IL10* gene into human MSCs and screened out IL10 highly overexpressing clones, which then underwent comprehensive assessments including general features, differentiation capabilities, and immunomodulatory properties of IL10 genetically modified MSCs (IL10-MSCs) according to the criteria of MSC-based therapeutic product. Afterwards, we applied IL10-MSCs to treat SCI in preclinical study and found IL10-MSC treatment significantly reinforced motor function recovery compared with naïve MSCs treatment. We further found that transplantation of IL10-MSCs drove the M2/M1 polarization of microglia/macrophages and reduced neuroinflammation in SCI mice. This study provides experimental evidence of IL10-modified MSCs reinforcing the therapeutic effects on SCI in compliance with quality criteria of cell-based therapeutic product for future clinical use.

## Materials and methods

### Isolation and culture of clinical-grade hUCMSCs

All procedures involving human subjects in this study were approved by the research ethics board of Nanjing Drum Tower Hospital (approval number GCP-SCP/17/2). All donors signed written informed consent to participate in this study. Clinical-grade hUCMSCs were used in this study that were greatly optimized in our previous research [7]. The entire process, including isolation, cultivation, identification, quality control, and storage, conformed to GMP quality standards. After evaluation, clinically qualified hUCMSCs derived from three umbilical cords of female newborns in second generation were mixed and resuscitated for following experiments to avoid individual heterogeneity [34].

### Preparation of clinical-grade IL10-MSCs

#### Vector construction

The human *IL10* gene (ref. sequence NM_000572.2) was synthesized by Sino Biological Co. LTD (Beijing, China) and confirmed by sequencing. The expression vector pCMV/hygro was designed for stable high-level expression in mammalian hosts. The vectors contain the following elements: human cytomegalovirus immediate-early (CMV) promoter for high-level expression in a wide range of mammalian cells, hygromycin resistance gene for selection of mammalian cell lines, and a Kozak consensus sequence to enhance mammalian expression. The IL10 recombinant vector was purified with QIAGEN Endofree plasmid kit (cat. no. 12362, QIAGEN, Hilden, Germany) and linearized with the restriction enzyme NruI (cat. no. 1168A, Takara Biomedical Technology Co., Ltd.) for electrotransformation.

#### Gene modification

The clinical-grade hUCMSCs at passage 2 (P2) were used for electrotransformation. To improve the efficiency of electrotransformation, we removed the skeleton structure of vector through linearization, and performed the electrotransformation using an optimized electrotransformation program in a CUY21EDIT II super multipulse in vivo and in vitro electroporator (BEX Co., Ltd.): pulse voltage (150 PpV), drive voltage (20 PdV), pulse time (10 ms), and number of cycles (20). The transfected cells were divided into two groups: blank control plasmid (NC) and recombinant *IL10* plasmid. After electrotransformation, the mixture was inoculated in 10-cm dish. After culturing at 37 °C and 5% CO_2_ for 24 h, cell status and mortality were evaluated, and then 100 μg/ml hygromycin was used for stable clone screening. After 14 days, cell colonies were observed under stereomicroscope (K-400L, Motic China Group Co., Ltd.) for expansion culture, and each colony represented a cell line.

### Identification and culture of IL10-MSCs

When each clone culture grew to 90% confluence, the supernatant was harvested and IL10 secretion level was detected using ELISA KIT (70-EK110/2-96, MultiSciences). Then, the stably expressing IL10 clones were expanded into P5–P6 generation and frozen as seeding cells. The negative control hUCMSCs were also obtained and cryopreserved in the same generation. Among multiple clones, we found that the 25# cell line had a good growth potential and secreted a high level of IL10. Then the 25# seeding cell line was resuscitated and passaged about one to two generations for subsequent experiments in vivo and in vitro.

### Quality assessment of IL10 genetically modified clinical-grade hUCMSCs

According to the guidelines of the Mesenchymal and Tissue Stem Cell Committee of the International Society for Cell & Gene Therapy (ISCT), hUCMSCs have three minimum definition criteria, including adhesion to plastic, specific cell-surface marker expression (CD105, CD73, CD90 positive cells ≥ 95%, CD14, CD34, CD45, CD19, and HLA-DR positive cells ≤ 2%), and multilineage differentiation potentials for adipogenesis, osteogenesis, and chondrogenesis.

#### Determination of surface marker expressions

A total of 2 × 10^5^ cells were harvested and resuspended in 100 μl 1× PBS, followed by incubation with the monoclonal antibodies CD34, CD45, CD90 [labeled with fluorescein isothiocyanate (FITC)], CD19, CD44, CD73, CD105, and HLA-DR [labeled with phycoerythrin (PE)] (BD, San Diego, CA, USA). After incubating for 30 min in the darkness at room temperature, cells were washed three times with 1× PBS and resuspended in washing buffer for flow cytometry analysis (BD FACSAria; NJ, USA). The data were analyzed with FlowJo V10 software.

#### Three-lineage differentiation potential assay

IL10-MSCs and control MSCs were seeded in 24-well culture plate at a density of 1 × 10^4^ cells per well in DMEM medium complemented with 10% fetal bovine serum (FBS) (10099141, Gibco). When the cells grew to 50–70% confluency, medium was replaced with adipose and osteogenic differentiation medium (Gibco, USA) to induce adipogenesis and osteogenesis, respectively. After 21 days, cells were fixed in 4% formaldehyde and stained with Oil Red O (Sigma-Aldrich, USA) and Alizarin Red S (Sigma-Aldrich, USA) to evaluate adipogenic and osteogenic differentiation, respectively. In addition, 2 × 10^5^ cells were centrifuged in a tube at 1200 rpm/min for 5 min, and chondrogenic differentiation medium (Gibco, USA) was added to the pellet after the supernatant was removed. After 21 days, the precipitate was fixed in 4% formaldehyde, dehydrated through serial ethanol dilutions, and then embedded in the optimal cutting temperature compound (OCT). The block was cut into 5-mm-thick sections and stained with Alcian Blue (Sigma-Aldrich, USA) to assess chondrogenic differentiation potential.

#### Safety evaluations

Studies have shown that, in the process of in vitro culture of MSCs, the probability of chromosomal abnormalities is 4%, and the tumorigenicity of stem cells is also a major safety hazard in clinical applications [35]. It is especially important to assess the genetic stability and tumorigenicity risk when gene-modified MSCs are applied to recipients; thus, we performed cell karyotyping to determine the genetic stability via Giemsa banding technique, and carried out tumorigenicity assays in immunodeficient mice to evaluate their therapeutic safety. Briefly, blank-vector-transfected MSCs and stable IL10-overexpressing MSCs at sixth generation were treated with 10 µg/ml colchicine solution (Roche, Basel, Switzerland) for 30 min at 37 °C. The cells are then fixed and spread according to standard procedures. Twenty metaphases were analyzed. In terms of tumorigenicity, male mice with severe combined immunodeficiency (SCID) were injected subcutaneously with 1 × 10^7^ IL10-MSCs at sixth generation or human embryonic stem cells (HESC) as a positive control [36], and monitored for tumor formation once a week for 4 months. After anesthesia, the mice were killed, and major organs were collected for hematoxylin–eosin (H&E) staining.

### RNA sequencing and differentially expressed gene analysis

For further safety evaluation, RNA-seq technology was conducted to test whether the introduction of IL10 into hUCMSCs activated tumor-related signaling pathways. Total RNA was extracted from control MSCs and IL10-MSCs, and 1 μg of total RNA was used to create a library for each sample. The libraries were sequenced on an Illumina HiSeq X Ten platform, and 150 bp paired-end reads were generated. RNA-seq and analysis were conducted by OE Biotech Co., Ltd. (Shanghai, China). Differentially expressed genes (DEGs) analysis was performed using the DESeq (2012) R package. The screening conditions of significantly differential expression for transcriptome were *p* value < 0.05 and fold change > 2 or fold change < 0.5. GO enrichment and KEGG enrichment analysis of DEGs were performed using DESeq R package based on hypergeometric distribution.

### SCI surgery and cell transplantation

SCI model was established according to our previously reported protocols [37]. In short, adult female C57BL/6J mice were anesthetized using 1.5–2% v/v isoflurane (VETEASY, RWD Life Science Co., Ltd, Shenzhen, P.R. China) and O_2_. Laminectomy was performed between T3 and T4 vertebrae, the spinal cord was completely transected, and the sham animals were exposed in the same way, but without causing damage to the spinal cord. Gelatin sponges were placed on the wound to achieve hemostasis. The muscle and skin were sealed with absorbable sutures (Vicryl, 3-0, Ethicon Inc., Cincinnati, OH, USA). The animals received 200 µL prewarmed saline by intraperitoneal injection, and then recovered on a heating pad for 3–6 h. Ceftriaxone sodium (50 mg/kg, drug license number H20043014; Youcare Pharmaceutical Group Co., Ltd., Corona, CA, USA) was injected intraperitoneally into animals every day to prevent infection, once a day for 3 days. During the entire experiment, the bladder of SCI mice was manually emptied twice each day.

A total of 56 adult female C57BL/6J mice (18–20 g) were randomly divided into four groups: sham, SCI + vehicle, SCI + MSCs, SCI + IL10-MSCs (*n* = 6–8 per group for behavioral assays and histology and immunostaining, *n* = 4–6 per group for flow cytometric analysis). A total of 1 × 10^6^ transplanted cells were suspended in 100 μl PBS, and cells were transplanted via tail vein 24 h post-SCI; meanwhile, the sham group was injected with equal volume of PBS. Thereafter, tail vein injection was performed every 2 weeks.

### Behavioral assays

The Basso Mouse Scale (BMS) was used to evaluate the locomotor functional performance and the progress of hind limb recovery of mice in all groups [38]. The BMS scale includes primary and subscoring systems, mainly to observe ankle movement, plantar placing or dorsal stepping, coordination, and trunk stability [39]. Two trained observers independently evaluated the animals in an open area for 4 min each week after injury. The performances of the left and right hind limbs were evaluated and averaged to generate the BMS score and subscores.

### Cell culture and treatments

The BV2 microglial cell line was cultured in DMEM (12100046, Gibco) supplemented with 10% FBS and 10,000 U/ml penicillin–streptomycin (15140122, Gibco). To evaluate the effect of IL10-MSCs on activation of microglia, we first collected the conditioned medium of MSCs and IL10-MSCs, and then inoculated BV-2 in a 12-well plate at 0.2 × 10^6^ cells per well. BV2 cells were cultured with DMEM/F12 supplemented with B27, 500 ng/ml LPS (L6143, Sigma-Aldrich), and 2.5 ng/ml IFN-γ (485-MI-100, R&D) for 24 h; the medium was harvested as M1 condition medium for following experiment. Medium only supplemented with B27 from BV2 cell culture was used as negative control.

NSCs were cultured from mouse embryo hippocampus. The hippocampus was dissected from embryos of female C57BL/6 pregnant mice (days 12–14) and digested with Accutase solution (A6964, Gibco) at 37 °C for 20 min. The hippocampus tissue was transferred into a new 50 ml centrifuge tube and was gently blown with pipette until tissue mass disappeared. The cell suspension was incubated in DMEM/F12 medium (Gibco) supplemented with 20 ng/ml epidermal growth factor (EGF), 20 ng/ml basic fibroblast growth factor (bFGF), 2% B27 (Invitrogen), and 1% penicillin–streptomycin. After two or three generations, the neutrospheres of NSCs were digested into single cells and resuspended in DMEM/F12 medium containing 10% FBS. Then, 3.5 × 10^5^ NSCs were seeded on a cover glass in a 12-well plate as the lower chamber. A total of 1 × 10^5^ MSCs or IL10-MSCs were seeded in the upper chamber of the trans-well insert. To analyze whether IL10-MSCs could antagonize the inflammatory cytokines’ adverse effects on viability and differentiation of NSCs in M1 condition medium, NSCs were co-cultured with MSCs or IL10-MSCs using a trans-well system in same plate at 37 °C for 5–7 days to detect neural differentiation, or co-cultured for 24 h to assay viability.

### Cell viability

Viability of NSCs co-cultured with MSCs or IL10-MSCs in LPS-activated BV2 derived condition medium was determined by Calcein-AM/PI Double Staining kit (C542, DOJINDO Molecular Technologies, Inc.). Following 24 h incubation, 500 μl of the combined LIVE/DEAD cell staining solution (2 µmol/l Calcein-AM and 4.5 µmol/l PI in DPBS) was added to each substrate and incubated with cells for 20 min at 37 ℃. Images were obtained using a fluorescence microscope (Leica Microsystems, Germany).

### Histology and immunostaining

Animals were deeply anesthetized and intracardially perfused with 4% paraformaldehyde at indicated time. The spinal cords were fixed in 4% PFA, soaked in 20% and 30% sucrose overnight, embedded in optimal cutting temperature (OCT) compound, and sectioned into 8-μm sections using a cryostat. Hematoxylin and eosin (H&E) staining was performed for general histological examination. For immunostaining, sections were permeabilized with PBS containing 0.5% Triton X-100 for 15 min. The tissue sections or cells were blocked with 5% goat serum for 1 h, and then incubated overnight at 4 °C with following primary antibodies. After washing three times with PBS, the corresponding second antibodies conjugated with Alexa Fluor 488 or Alexa Fluor 568 (1:500, Invitrogen, Carlsbad, CA, USA) were probed for 1 h at room temperature. Finally, the coverslips were washed with PBS three times and mounted using 4′-6-diamidino-2-phenylindole (DAPI) (ab104139, Abcam) solution.

The following primary antibodies were used: anti-glial fibrillary acidic protein antibody (GFAP) (1:500, ab7260, Abcam, Cambridge, UK), anti-neurofilament antibody (NF) (1:200, ab3966, Abcam, Cambridge, UK), βIII tubulin (Tuj-1) (1:500, ab7751, Abcam, Cambridge, UK), CD11B/integrin alpha M polyclonal antibody (1:200, 66519-1-Ig, Proteintech Group, Inc.), arginase 1 polyclonal antibody (1:300, 16001-1-AP, Proteintech Group, Inc.), and iNOS polyclonal antibody(1:20018985-1-AP, Proteintech Group, Inc.). The targeted marker-positive cells in each visual field were counted under Leica DMi8 confocal laser scanning microscope. Five or six randomly chosen fields from the lesion epicenter in each section of at least ten sections from each sample in all groups were examined.

### Flow cytometric analysis

#### Spinal cord samples

At day 14 after SCI, the damaged spinal cord (from 2.5 mm caudal to 2.5 mm rostral at the lesion site) was surgically dissected and then dissociated with 175 U/ml collagenase at 37 °C for 1 h. The cells were washed in DMEM containing 10% FBS and filtered through a 40-µM nylon cell strainer (352340, BD Falcon) under centrifugation to remove tissue debris and obtain a single cell suspension. Before performing flow cytometry immunostaining, each sample was counted to maintain a concentration of 1.0 × 10^6^ cells per 100 µl. Cells were incubated for 30 min at room temperature with CD45-FITC (11-0451-82, eBioscience), CCR2-BV510 (747970, BD), FVS-APC-Cy7A(565388, BD), and CD11b-PerCP-Cy5.5 (45-0112-82, eBioscience), then were fixed and permeabilized for intra- and extracellular staining with a Fixation & Permeabilization Kit (FMS-FP0100,Fcmacs Biotech Co., Ltd.) and incubated for 30 min at room temperature with the following fluorescent antibodies: iNOS-PE (12-5920-82, eBioscience) and arginase 1 (Arg1)-EF450 (48-3697-82, eBioscience). FVS staining was added to exclude dead cells. CD45^hi^/CD11b^lo^ cells were designated hematogenous macrophages, and CD45^lo^/CD11b^hi^ cells were designated activated endogenous microglia [40].

BV2 cells were seeded onto six-well plates (5 × 10^5^ cells per well), and cell culture was performed as described above. BV2 cell markers CD11b and F4/80 were analyzed by flow cytometry, and activated microglia gating was further evaluated by incubating with activation markers of Arg1 and iNOS. After 2 days of culture, cells were trypsinized, then incubated with F4/80-FITC (11-4801-85, eBioscience) and CD11b-PerCP-Cy5.5 for 30 min at ambient temperature. After washing twice with PBS, cells were fixed, permeabilized, and incubated for 30 min at room temperature with the fluorescent antibodies of iNOS-PE and Arg1-EF450.

Flow cytometry was performed immediately with a FACSAria (BD, NJ, USA) using forward scatter to further eliminate any cellular debris from analysis. Living singlets were gated using FSC-W/FSC-H. Data were analyzed using FlowJo Version 10 (Tree Star, Ashland, OR, USA).

#### RNA extraction and qRT–PCR

Total RNA was extracted from spinal cord or cells with Trizol reagent (Invitrogen), and 1 μg of RNA was used to synthesize cDNA (HiScript II 1st Strand cDNA Synthesis Kit, Vazyme). Quantitative PCR was performed using ChamQ TMSYBR qPCR Master Mix (Vazyme) in an ABI Quant Studio 6 qPCR system (Applied Biosystems), under the following conditions: 95 °C for 5 min followed by 40 cycles of 95 °C for 15 s and 60 °C for 30 s. *GAPDH* was used as the housekeeping gene for normalization. The target genes from the experimental groups were compared with those from the control group using the 2^−△△CT^ method. The following primers were used: *iNOS*-forward: CCCTTCAATGGTTGGTACATGG, *iNOS*-reverse: ACATTGATCTCCGTGACAGCC; *Arg1*-forward: CTCCAAGCCAAAGTCCTTAGAG, *Arg1*-reverse: GGAGCTGTCATTAGGGACATCA; *CD11b*-forward: CCAAGACGATCTCAGCATCA, *CD11b*-reverse: TTCTGGCTTGCTGAA TCCTT; *IL1β*-forward: CGTGCTGTCGGACCCATATGAG, *IL1β*-reverse: GCCCAAGGCCACAGGTATTT; *IL10*-forward: GCTGAGGCGCTGTCATCGATTT, *IL10*-reverse: GGCCCTGCAGCTCTCAAGTGT.

### Statistical analysis

All values were expressed as mean ± standard derivation (SD). Differences between groups were examined for statistical significance using one-way factorial analysis of variance with Tukey’s multiple comparison tests. GraphPad prism version 5.0 (GraphPad Software, San Diego, CA, USA) was used to estimate significance and produce graphs. *p* < 0.05 denoted the presence of significant difference.

## Results

### Preparation of clinical-grade IL10-MSCs

**Fig. 1:**
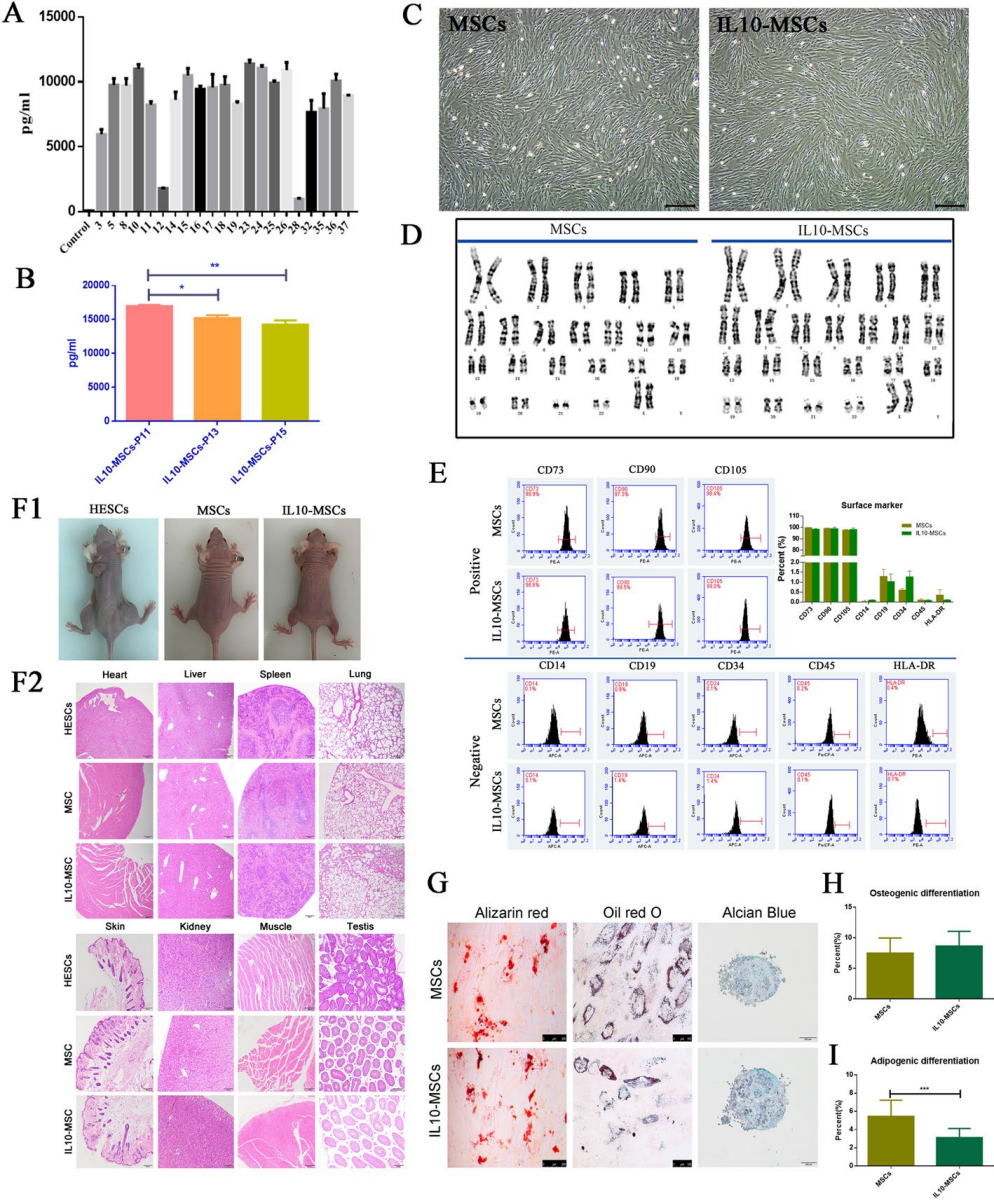
Systematic quality evaluation of clinical-grade IL10-MSCs. **A** The IL10 protein level in the supernatant of different IL10-MSCs clones (P5-6). MSCs with blank vector transfection were the control. **B** With the increase in the number of passages, the IL10 secretion gradually decreased in IL10-MSCs, but still sustained a high level. **C** Morphological characterization of IL10-MSCs (P6) compared with naïve MSCs (P6) under light microscopy. **D** Representative karyotype analysis. Control MSCs (left, P6) and IL10-MSCs (right, P6) both had normal karyotypes. **E** Flow cytometric analysis showed IL10-MSCs were positive (> 95%) for mesenchymal lineage markers (CD73, CD90, and CD105) and negative (< 2%) for hematopoietic and endothelial markers (CD14, CD19, CD34, CD45, and CD3) as well as for HLA-DR, in accordance with the criteria of clinical-grade MSCs. **F1–2** Tumorigenesis analysis showed that there was no tumor formation observed in any SCID mice injected with IL10-MSCs, but tumor was formed in positive control SCID mice with HESCs transplantation. Further H&E staining showed no infiltration of tumor cells at the cell injection site and main organs after IL10-MSC injection. **G**–**I** Differentiation potential of IL10-MSCs in adipocytes, osteocytes, and chondrocytes stained with Oil red O, Alizarin Red, and Alcian Blue, respectively. IL10-MSCs maintained multiple lineage potential, but with downregulated adipogenic differentiation capability

A total of 37 clones were identified, and their IL10 expression levels in culture supernatant (passage 6, P6) were measured by ELISA. The IL10 transduction efficiency was as high as 56.8%, and the IL10 secretion levels of different clones are shown in Fig. 1A (negative clones not shown). We selected 25# clone with high expression of IL10 and fast growth among these positive clones for follow-up experiments. To determine stability of IL10 expression in IL10-MSCs, we detected IL10 expression levels in culture supernatants of P11, P13, and P15 generations of 25# clone via ELISA. Results showed that, although IL10 secretion levels somewhat decreased as the number of generations increased, they still maintained a high expression level (Fig. 1A, B).

### Systematic quality evaluation of clinical-grade IL10-MSCs

For 25# clone, *IL10* gene modification did not alter the long fusiform morphology and surface marker expression profiles of MSCs. As shown in Fig. 1C, the cell morphology of naïve MSCs (P6) before electroporation was similar to that of IL10-MSCs (P6) after electroporation. Surface marker expression analysis showed that IL10-MSCs positively expressed surface markers CD73, CD90, and CD105 (more than 95%), and negatively expressed surface markers CD19, CD34, CD11b, CD 45, and HLA-DR (less than 2%), like the naïve MSCs (Fig. 1E). Karyotype analysis was performed to assess genetic stability of IL10-MSCs and MSCs. The results showed that, like naïve MSCs (P6), IL10-MSCs (P6) had a normal karyotype, including normal number (46, XX), morphology, length, size, and centromere position of chromosomes, without any abnormality such as deletion, reduplication, inversion, translocation, insertion, or ring chromosome (Fig. 1D).

The tumorigenicity risk of transplanted gene-modified MSCs is an important safety concern. IL10-MSCs were injected subcutaneously into both necks of severe combined immune deficiency (SCID) mice to investigate tumor formation during a long-term observation period of 4 months. As shown in Fig. 1F, human embryonic stem cells (HESCs) were transplanted into SCID mice as the positive control and tumor formations were observed within about 4 weeks after transplantation. In contrast, we did not observe tumor formation in any SCID mouse with MSC or IL10-MSC injection. H&E staining further showed no tumor cell infiltration at the cell injection site or major organs, including testis, lung, heart, muscle, spleen, liver, kidney, and skin. Whether gene-modified MSCs maintain the differentiation potential of multiple lineages is another important concern. We analyzed the differentiation potentials of IL10-MSCs including osteogenic, adipogenic, and cartilage differentiation by Alizarin Red, Oil red O, and Alcian Blue staining, respectively. As shown in Fig. 1G, H, IL10-MSCs maintained multilineage differentiation potential like naïve MSCs, but the adipogenic differentiation capability was downregulated by IL10 modification.

### Introduction of IL10 to human MSCs did not significantly alter tumor‑associated signaling pathways

**Fig. 2:**
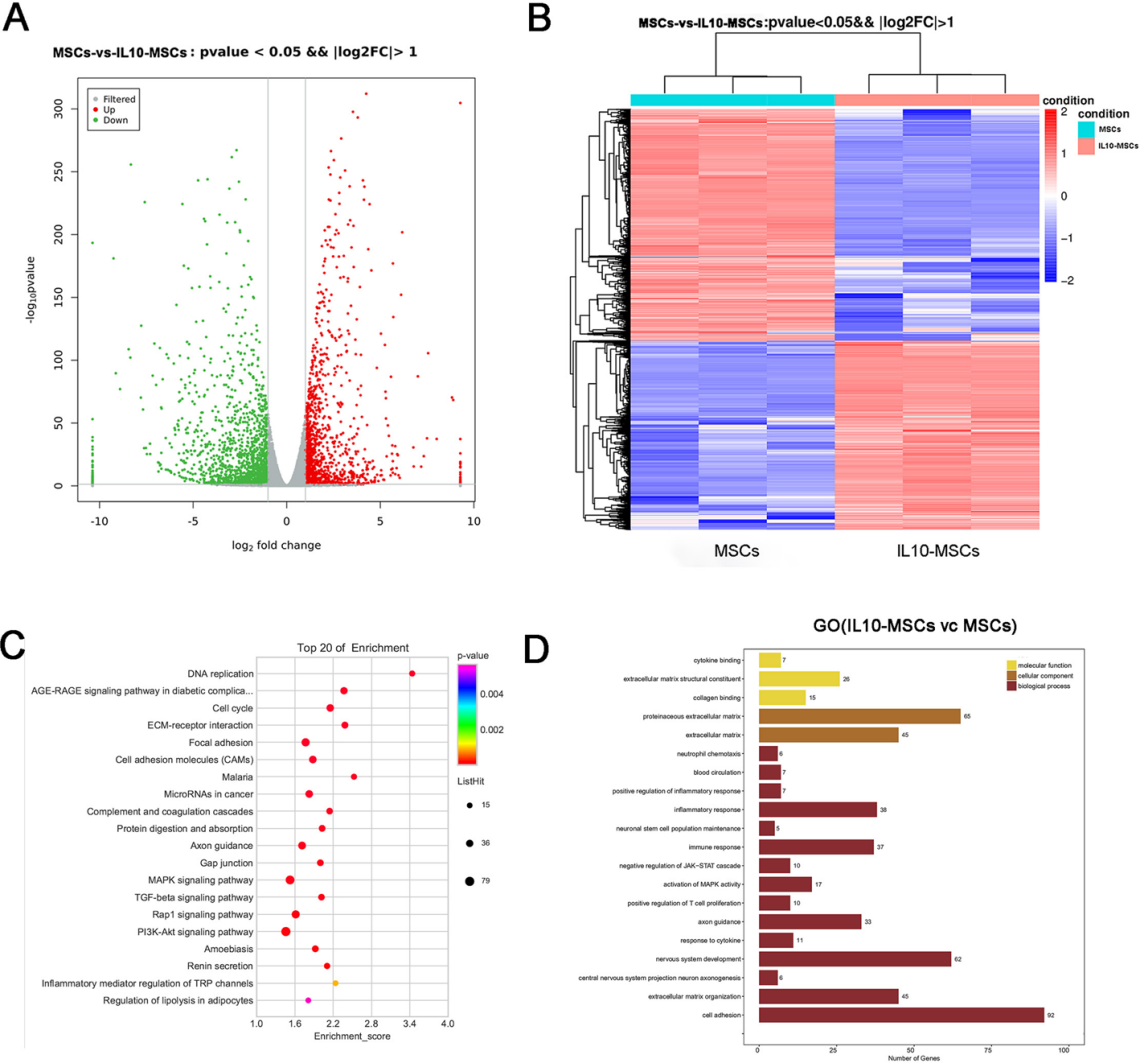
Transcriptome and analysis of IL10-MSCs and MSCs by RNA sequencing (RNA-seq). **A** Volcano diagram of differentially expressed genes. Gray indicates the genes with insignificant difference, and red and green those with with significant difference. **B** Heat map of all differentially expressed genes between IL10-MSCs and MSCs with a *p*-value of < 0.05. Red and blue represent highly and weakly expressed genes, respectively, between IL10-MSCs and MSCs. **C** The top 20 KEGG pathway analysis. **D** GO analysis of significantly enriched genes

Although we did not observe tumor formation on tumorigenicity risk assays after IL10-MSC transplantation in SCID mice, we still need to determine whether exogenous gene introduction activated tumor-associated signaling pathways. We compared gene expressions between MSCs and IL10-MSCs by high-throughput RNA sequencing. The data in Fig. 2 are from three replicates of the same clone IL10-MSCs (25#) and MSCs. Gene ontology (GO) enrichment analysis suggested that most differential genes were associated with immune response, inflammatory response, and cell migration (Fig. 2D), which was consistent with the biological functions of IL10 including anti-inflammatory and immune regulation (Fig. 2D) [41]. Importantly, although KEGG pathway analysis showed that cancer-related pathways were increased slightly, the enrichment score was only slightly increased, which was mainly due to genes related to cell proliferation, while tumor biomarker genes did not change significantly. Therefore, the introduction of *IL10* gene into MSCs did not remarkably activate tumor-related genes, and it is considered safe for clinical use.

### IL10-MSC treatment improved functional recovery and reduced lesion size

**Fig. 3:**
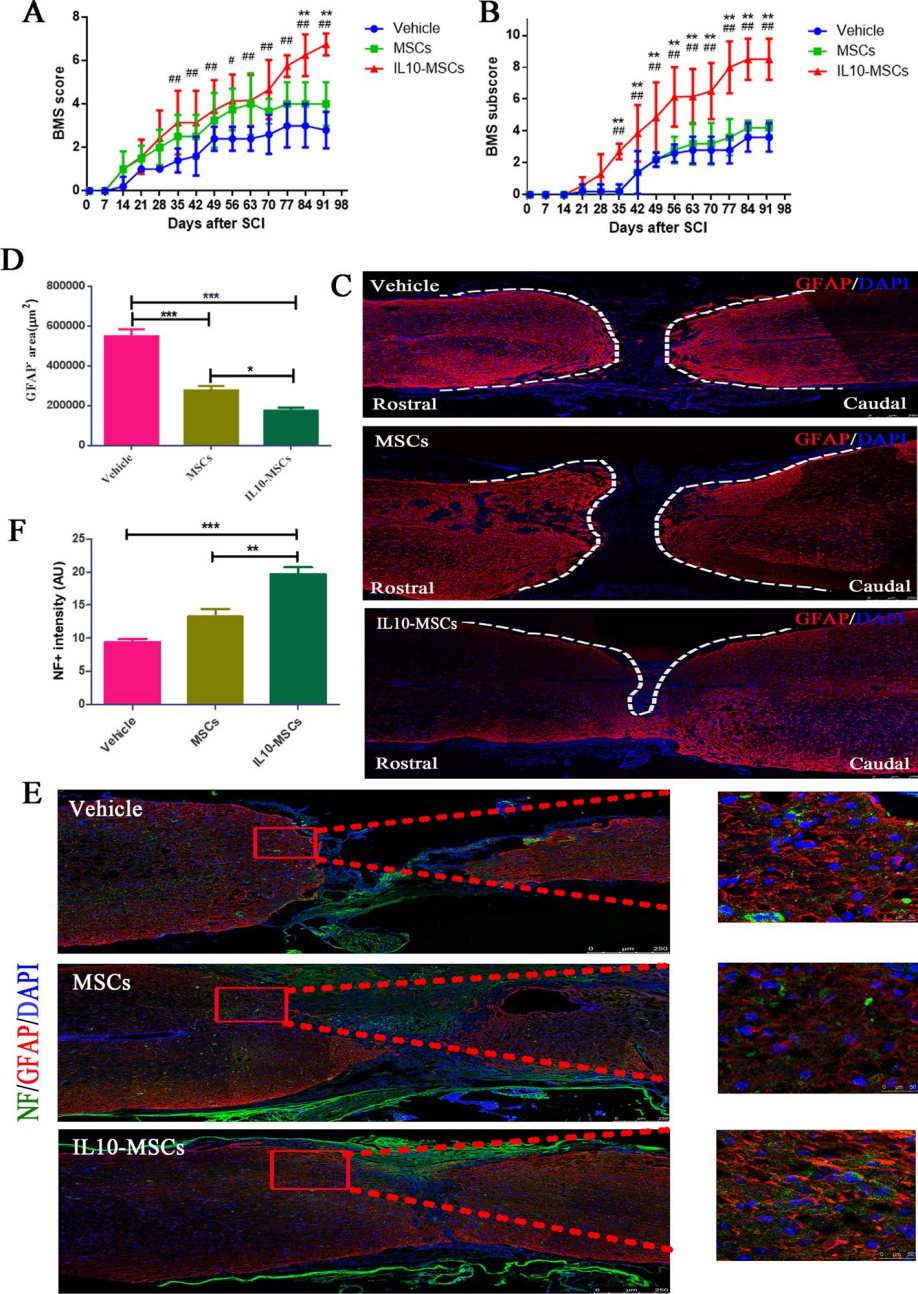
Transplantation of IL10-MSCs increased functional recovery in mice following SCI. **A**, **B** IL10-MSC treatment showed a statistically significant recovery over time with coordinated stepping (BMS ~ 7, **p < 0.01) and improved limb movement (BMS subscores ~ 7, **p < 0.01) at day 91 post-injury compared with MSC group. **C**, **D** Lesion sizes were quantified by immunostaining against GFAP, as depicted by the dotted white line. Image analysis revealed a significant decrease in mice treated with IL10-MSCs, compared with vehicle (***p < 0.001) and MSC groups (*p < 0.05). **E**,** F** Quantification of NF-positive staining revealed a greater increase in IL10-MSC group compared with vehicle (***p < 0.001) and MSC groups (**p < 0.01) (Scale bars, 250 and 50 μm). Data represent mean ± standard error of the mean (SEM) (ANOVA, ***p < 0.001, **p < 0.01, *p < 0.05. *n* = 6–8 mice per group)

After screening out positive IL10-MSC clones and performing systematic quality assessments, we investigated whether IL10-MSC transplantation could promote better functional recovery in C57BL/6 mice following SCI. Considering the convenient clinic application in future, we performed repeated intravenous injections to obtain a better therapeutic effect. Mice suffering from SCI were treated with vehicle (0.9% NaCl solution), control MSCs, and IL10-MSCs by intravenous injection every 2 weeks, respectively. The BMS score and BMS subscore were evaluated at indicated times (Fig. 3A, B). As expected, from day 2 (D2) to week 2 (W2), mice in all groups had low scores, without statistically significant difference among all groups. At W6, both MSC and IL10-MSC groups displayed a significant improvement in functional recovery, higher than vehicle group (*p* < 0.01) (Fig. 3A). Furthermore, the IL10-MSC group evidently showed better therapeutic improvement than the MSC group. Compared with the MSC group, the BMS scores in the IL10-MSC group increased to 6.2 ± 0.84 and 6.6 ± 0.55 at W12 and W13, respectively (**p < 0.01).

An important aspect of motor recovery after SCI is whether animals have coordinated movements between the fore and hind limbs [42]. With the recovery in SCI mice, the pedaling of the hind limbs gradually rehabilitated, but the pedaling frequency of the forelimbs and hind limbs was usually asymmetric. The BMS subscore further quantified the aspects of fine motor recovery, including plantar stepping, paw/tail positions, and trunk instability [39]. At W6, the BMS subscores in the MSC and IL10-MSC groups were significantly higher than in the vehicle group (*p* < 0.01). As evident from Fig. 2B, the BMS subscore in the IL10-MSC group was higher than that in the other two groups. Immunofluorescence staining against glial fibrillary acidic protein (GFAP) and neurofilament (NF) (Fig. 3C and E) was carried out to analyze the lesion size and neuronal regeneration at W13 post-SCI [43]. The quantification of the lesion sizes revealed a significant decrease in the IL10-MSC group compared with the MSC and vehicle groups (Fig. 3C), accompanied by a 36.9% decrease in the IL10-MSC group compared with the MSC group (Fig. 3D; *p < 0.05). Quantification of NF intensity at the epicenter of the injured spinal cord showed there were more NF-positive cells in the IL10-MSC group compared with the other two groups (Fig. 3F).

### Transplantation of IL10-MSCs facilitating M2 activation

**Fig. 4:**
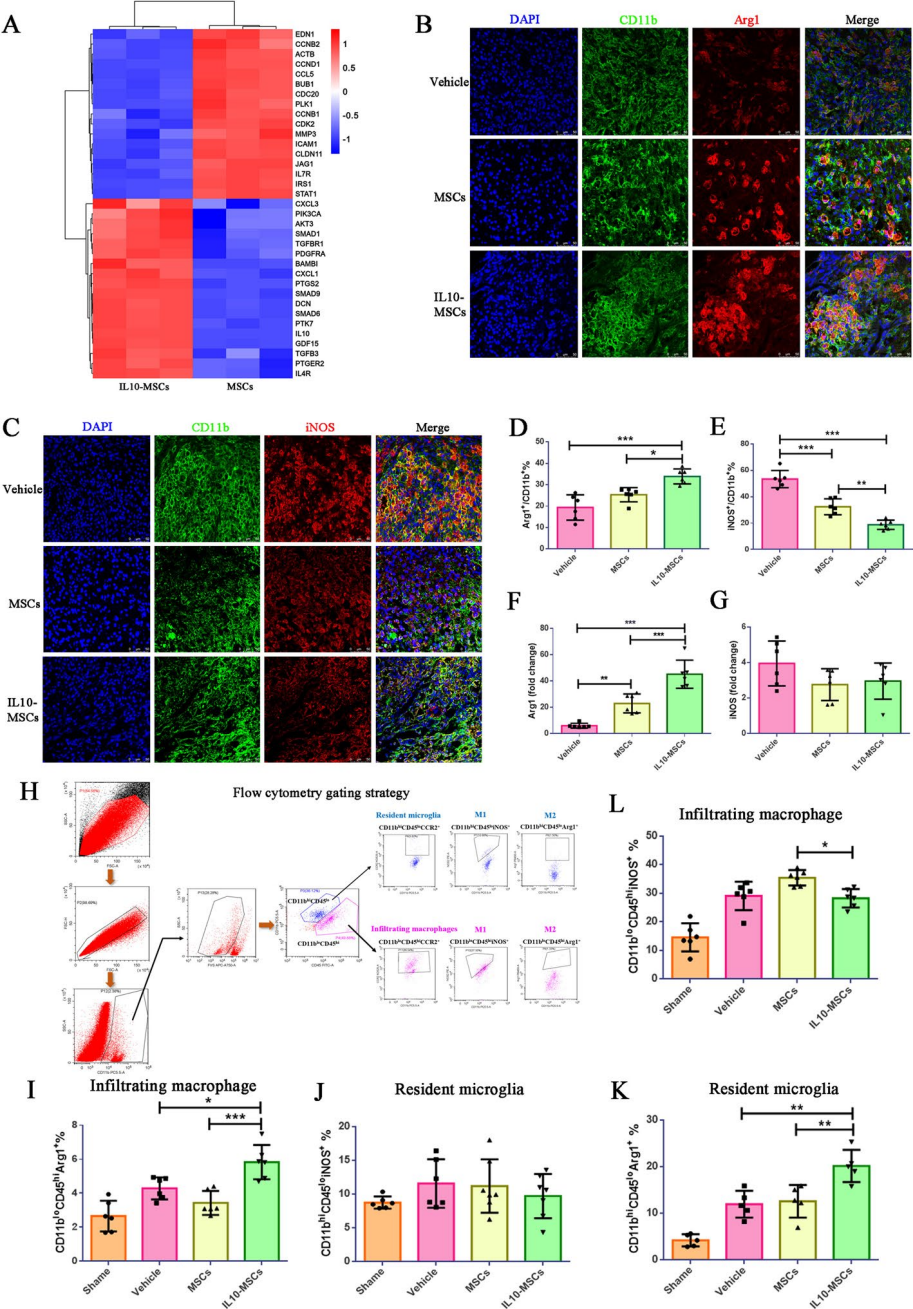
Transplantation of IL10-MSCs alternatively activated M2 microglia/macrophages at the lesion site. **A** Heat map of the most differentially expressed genes involved in regulating immune and macrophage polarization between IL10-MSCs and MSCs. The alternatively activated M2 and M1 microglia/macrophages at lesion regions were identified by immunostaining against CD11b/Arg1 and CD11b/iNOS, respectively. **B**, **D** At W2 after SCI, IL10-MSC treatment significantly increased the ratio of Arg-1^+^ cells to CD11b^+^ cells compared with MSC graft and vehicle control. **C**, **E** The ratio of iNOS^+^ cells to CD11b^+^ cells in the IL10-MSC group were significantly decreased compared with the other two groups. **F**, **G** The mRNA expression of iNOS and Arg1 was assessed in injured sites. Consistent with immunostaining results, IL10-MSCs treatment significantly improved the mRNA expression of Arg-1 at lesion site compared with the other two groups, and decreased mRNA expression of iNOS, compared with control group. **H**–**L** Cytometry markers and gating strategy were performed to distinguish the resident microglia and infiltrated macrophage at lesion site. **H** Gates to exclude debris and cell aggregates in FSC-A/SSC-A and FSC-A/FSC-H plots. Representative flow cytometry gating strategy correspond to resident microglia (CD11b^hi+^/CD45^lo^/CCR2^−^) and infiltrated macrophages (CD11b^lo^/CD45^hi^/CCR2^+^). Activated microglia/macrophage gating was further stratified by incubation with activation markers Arg1 and iNOS. The area outside the purple line represents the resident microglia, and the area within the red line represents the infiltrated macrophages. **I**, **J** M1 and M2 macrophages were identified according to iNOS and Arg1 expression. Graph shows the ratio of M1 to M2 infiltrated macrophages at the injured spinal cord. **K**, **L** Ratio of M1 to M2 resident microglia cells at injured spinal cord. Data represented as mean ± SEM; ***p < 0.001, **p < 0.01, *p < 0.05; *n* = 6–8 per group (**B**, **D**), *n* = 4 per group (**I**–**L**); Scale bars, 50 um (**B**, **D**)

Interestingly, when we conducted the safety evaluation of IL10-MSCs, we found that the targets were mainly enriched in macrophage polarization signaling pathway according to the KEGG (Fig. 3C), suggesting that this is one of the mechanisms of IL10-MSC therapy in SCI. Analysis of DEGs showed that the introduction of *IL10* gene enhanced immune and anti-inflammatory responses of MSCs. Among them, TGF-β regulates lipopolysaccharide-stimulated macrophage M2-like polarization via the Akt/FoxO1 pathway [44]; meanwhile, IL10 is a well-known anti-inflammatory cytokine that could promote the transition of M1/M2 macrophages. IL10 and TGF-β were expressed more abundantly by IL10-MSCs (Fig. 4A), indicating their possible regulatory role in macrophage polarization.

In the first attempt to understand the mechanism by which IL10-MSCs graft obtained better therapeutic outcomes, including reducing lesion size, increasing neuronal regeneration, and improving locomotion recovery, we investigated the phenotypic transition of alternately activated microglia/macrophages. We characterized the alternatively activated microglia/macrophage at the lesion site by immunostaining against CD11b, Arg1, and iNOS, respectively. The cells expressing CD11b, Arg1, and iNOS from 5 mm caudal to 5 mm rostral spinal cord at the lesion site were quantified. Double immunostainings against Arg1 and CD11b were used to identify the M2 phenotypic macrophages/microglia, and double stainings against iNOS and CD11b were used to identify the M1 phenotypic macrophages/microglia. Compared with control, no significant difference was observed in the amount of microglia/macrophage (CD11b positive cells) at the injured site in the MSC and IL10-MSC groups (Additional file 1: Fig. S3). We quantified the number of stained cells around the epicenter lesion and observed that the number of Arg1 and CD11b double-positive cells was significantly increased in IL10-MSCs group, compared with the MSC and control groups (Fig. 4B, D, *p < 0.05, ***p < 0.001), and the populations expressing iNOS at the lesion site regions were markedly decreased, compared with the other two groups (Fig. 4C, E). These data strongly indicate that the treatment of IL10-MSCs significantly increased the M2 phenotype of an alternatively activated microglia/macrophage at the lesion site, compared with MSC treatment and vehicle control. Next, we evaluated the M1/M2 polarization of macrophages/microglia after SCI by determining the mRNA expression of M1 markers (*iNOS*) and M2 markers (*Arg1*) in the injured spinal cord. Results showed IL10-MSC treatment significantly increased the mRNA expression of *Arg1* at day 14 after SCI, compared with the other two groups (***p < 0.001) (Fig. 4F), consistent with the results of immunofluorescence staining.

To investigate the cellular inflammatory response at lesioned spinal cord after IL10-MSC transplantation, we evaluated the activation of resident microglia and recruited macrophages at the lesion site at W2 after injury. In mice following injury, infiltrating macrophages and resident microglia were phenotypically and functionally heterogeneous, and the two kinds of immune cells could be distinguished according to CCR2 expression [45]. Given the extent of CD45 and CD11b expression, the resident microglia (CD11b^hi^/CD45^lo^/CCR2^−^) and infiltrating macrophages (CD11b^lo^/CD45^hi^/CCR2^+^) in the injured spinal cord were distinguished by FACS analysis (Fig. 3H–L) in accordance with previous study [46]. We found that there was no difference in the amount of microglia and recruited macrophages among all groups at day 14 after SCI in the injury site, as demonstrated by dot plots and quantitative analyses (Additional file 1: Fig. S3). Then, M1 cells and M2 cells were identified on the basis of the expression levels of iNOS and Arg1 by FACS analysis, respectively [47, 48]. The results showed that IL10-MSC treatment significantly improved the ratio of M2 resident microglia and infiltrated macrophages, compared with the other two groups (Fig. 4J, L). In contrast, IL10-MSC treatment only slightly decreased the ratio of M1 infiltrating macrophages and resident microglia, without statistical significance among the three groups. These results demonstrated that M2 activation was facilitated in resident microglia and infiltrated macrophages in injured spinal cord by IL10-MSC treatment.

### IL10-MSCs modulate the M2/M1 polarization of BV2 cells induced by LPS + IFN-γ

**Fig. 5:**
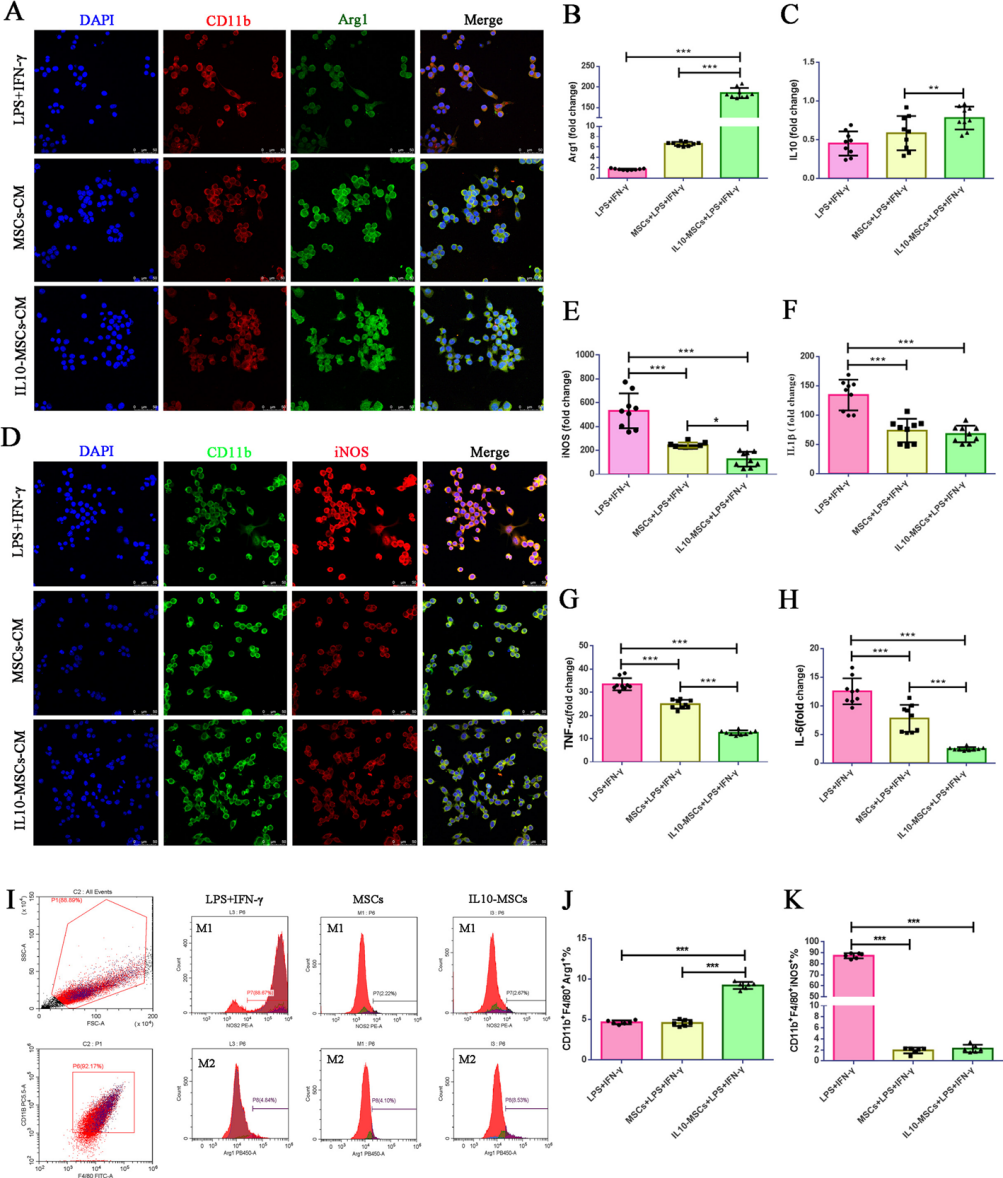
IL10-MSCs induced M2/M1 polarization of BV2 cells upon LPS and IFN-γ challenges. **A**, **D** Immunofluorescence staining was performed to assess Arg1 and iNOS expression in BV2 upon LPS and IFN-γ challenges in the presence of MSC- or IL10-MSC-derived CM. **B**, **C** IL10-MSC-derived CM obviously increased the mRNA expression of M2 markers (*Arg1* and *IL10*). **E**–**H** IL10-MSC-derived CM significantly decreased the mRNA expression of M1 markers (*iNOS*, *TNF-α*, *IL1β*, and *IL-6*) by qPCR. **I**–**K** FCA analysis was perform to investigate the percentage of M1 (CD11b^+^/F4/80^+^/iNOS^+^) and M2 (CD11b^+^/F4/80^+^/Arg1^+^) BV2 cells. (*n* = 6–8 per group, *p < 0.05, **p < 0.01, and ***p < 0.001)

Distinct inflammatory entities are known to trigger various macrophage profiles. We then attempted to determine the main spectra of microglia/macrophages upon IL10-MSC transplantation. BV2 cells, a mouse microglia cell line, were stimulated with LPS and recombinant IFN-γ, established factors that regulate M1 phenotype of macrophages [49]. We speculated that IL10 signaling might promote the M2/M1 transition of microglia/macrophages. MSCs and IL10-MSCs were cultured at 1 × 10^6^ cells in T75 flask for 48 h, and the conditioned mediums (CM) were harvested for following experiments. The BV2 cells were cultured with CM derived from MSCs or IL10-MSCs in the presence of LPS plus IFN-γ for 48 h. Immunostaining showed that MSC-derived CM and IL10-MSC-derived CM markedly decreased the M1 polarization of BV2 cells, at the same time promoting the M2 polarization of BV2 cells (Fig. 5A, D). At the same time, BV2 cells treated with LPS + IFN-γ displayed numerous lamellar and elongated filopodia, similar to M1 polarized microglia. When co-cultured with IL10-MSC-derived CM, the percentage of BV2 cells with lamellar processes was markedly decreased and most cells turned back to compact round M2-polarized microglia. For further quantifying the M2/M1 transition, the corresponding marker expressions were investigated by qPCR. IL10-MSC-derived CM significantly decreased the mRNA expression of *iNOS*, *TNF-α*, *IL1β*, and *IL-6* regarding M1 markers compared with control (Fig. 5E–H), while obviously increasing the mRNA expression of *Arg1* and *IL10* regarding M2 markers compared with the other two groups (Fig. 5B, C). Consistent with above experiments, FACS analysis further demonstrated that IL10-MSC-derived CM treatment significantly increased the percentage of CD11b^+^/F4/80^+^/Arg1^+^ BV2 cells upon LPS and IFN-γ challenges and reduced the percentage of CD11b^+^/F4/80^+^/iNOS^+^ population (Fig. 5I–K).

### IL10-MSCs rescued the inhibition of neuronal differentiation and death of NSCs upon activated BV2 cells

**Fig. 6:**
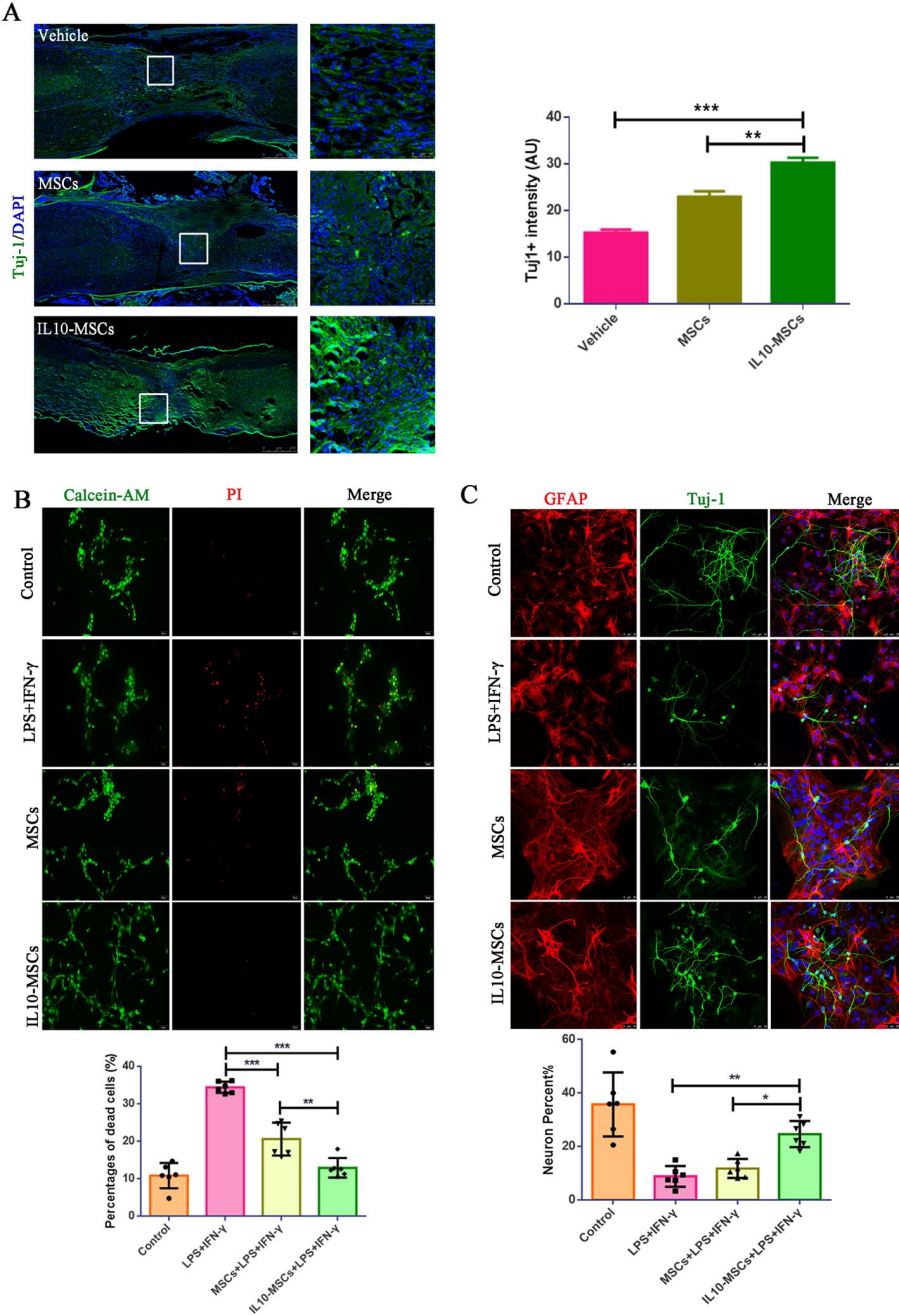
The grafting of IL10-MSCs reduced the inhibition of neuronal differentiation and death of NSCs induced by activated BV2 cells. **A** Quantification of Tuj-1 staining revealed a significant increase in Tuj-1-positive cells in IL10-MSC group compared with vehicle (***p < 0.001) and MSC groups (**p < 0.01) via optical density analysis. **B**, **C** The rate of death and neural differentiation of NSCs after being co-cultured with IL10-MSCs and MSCs in the presence of BV2-derived CM. Live and dead cells were stained green and red, respectively. The percentage of neurons derived from NSCs was quantified by Tuj-1 immunofluorescent staining. Results revealed that IL10-MSCs could rescue the neuronal differentiation inhibition of NSCs induced by activated BV2 cells. Scale bars, 250 and 50 μm. Data are expressed as mean ± SEM (*n* = 6), *p < 0.05, **p < 0.01, ***p < 0.001

From the results above, we concluded that IL10-MSC treatment inhibited the M1 subtype and preserved the M2 phenotype of microglia/macrophage at injured spinal cord. The inflammatory cytokines secreted from M1-activated microglia also inhibited the neuronal differentiation of NSCs and decreased the cell viability [50]. Immunofluorescence staining against Tuj-1, a neuronal marker, showed that the IL10-MSC group had more Tuj-1-positive cells in lesion section than the control and MSC groups (Fig. 6A). We wondered whether IL10-MSCs could rescue the harmful effects of activated M1 microglia/macrophages on NSCs. Calcein-AM and immunostaining against anti-Tuj1 (neuron marker) and GFAP (astrocyte marker) antibodies was performed to assay the survival and neural differentiation of NSCs, respectively. BV2 conditioned medium treated with LPS + IFN-γ markedly inhibited the neuronal differentiation of NSCs. However, the co-culture of IL10-MSCs with BV2 cells rescued the inhibition of neuronal differentiation derived from NSCs, compared with the MSC-co-culture group in vitro (Fig. 6C). Calcein-AM and PI staining indicated that BV2-derived CM caused an increase in the death of NSCs, but co-culture with IL10-MSCs reduced the death induced by BV2-derived CM (Fig. 6B).

## Discussion

SCI causes the loss of connection between the brain and the peripheral nervous system, resulting in temporary or permanent changes in the normal motor, sensory, and autonomic functions of the spinal cord. SCI can also affect most bodily functions, including the peripheral immune system, and induce many dysfunctions of breathing and intestines [51–53]. There is currently no effective treatment that can reach complete functional recovery after severe SCI. Because MSCs have immunomodulatory effects, and produce anti-apoptotic molecules as well as nutritional factors through paracrine action to exert neuroprotective functions, they have been widely used in therapy of SCI in animal model and clinic trials [54, 55]. However, until now, no MSC-based therapy could completely rehabilitate patients with SCI, owing to the limited therapeutic activity of naïve cells. Therefore, clever strategies of cell-based treatment need to be developed to improve the therapeutic effect, including gene modification, optimizing the type, number, and delivery method of seeding cells. In the present study, we introduced *IL10* gene into human umbilical-cord-derived MSCs to establish highly overexpressing IL10-MSCs strains in compliance with GMP guidelines. For safety evaluation, we did not observe tumorigenesis in SCID mice after subcutaneous injection of IL10-MSCs. Furthermore, RNA-seq analysis showed that tumor-associated signaling pathways were not induced in IL10-MSCs. To our knowledge, this is the first systematic quality and safety evaluation of gene-modified human MSCs, and IL10-MSCs are safe for clinic use. After comprehensive evaluations according to the requirements of MSC-based therapeutic products, the qualified IL10-MSCs were applied to mice with completely transected SCI. We observed the IL10-MSC treatment had better therapeutic outcomes, including reinforcing locomotor improvement, decreased lesion volume, and preservation of neurons, compared with control MSCs. In addition, we found that IL10-MSC transplantation promoted the transition of microglia/infiltrated macrophages M2/M1 ratio at the injured sites. Interestingly, IL10-MSC treatment also mediated the spectra of peripheral immune system, such as increasing the percentage of Treg and Th2 cells and reducing the percentage of Th1 cells. In vitro, IL10-MSCs modulated the M2 polarization of BV2 cells induced by LPS + IFN-γ. IL10-MSCs rescued the inhibition of neuronal differentiation and death of NSCs upon activated BV2 cells. Taken together, our findings indicate that IL10-MSCs could meet the requirements of cell-based therapeutic product and be an applicable cell-based therapy strategy for SCI, with more therapeutic advantages than control MSCs.

Immune responses play a crucial role in the initiation, progress, and regeneration processes of SCI. Anti-inflammation strategy using anti-inflammatory cytokines is a promising method for treating SCI. In recent years, several studies have reported that the well-known anti-inflammatory cytokine IL10 could achieve intraspinal sparing of neurons and axons associated with improved locomotor function after thoracic SCI [56, 57]. Accumulating evidence indicates that combination therapy of MSCs and IL10 protein could have synergistic effects to reduce inflammation in various diseases such as experimental autoimmune encephalomyelitis [58], ischemia–reperfusion injury in the lung [59], and ischemic stroke [60, 61]. However, owing to the short half-life of IL10 protein (about 1–2 min) in vivo and high costs, their prolonged therapeutic effects in clinical trials have been greatly limited [41]. We speculated that long-term maintenance of IL10 at effective dose at injured sites could result in better therapeutic outcomes. MSCs have chemotactic properties and are capable of migrating to injured sites. Thus, they are very suitable as carriers for delivering desired key target proteins such as IL10 or neurotrophic factors that are potentially beneficial to SCI recovery. In our study, we successfully established clinic-grade IL10-MSC strains that resulted in fine motor function recovery when applied to mice with SCI through repeated intravenous infusions. The IL10 protein could be detected in serum by ELISA and maintained for 72 h after intravenous infusion. In addition, GFP-labeled IL10-MSCs could be detected by immunostaining at injured spinal cord (Additional file 1: Fig. S3), consistent with a previous report by Bruna dos Santos Ramalho et al., who found that intravenously injected MSCs were able to migrate to the injured spinal cord and survive in the new milieu within a few days to 1 week [62]. These results indicate that IL10-MSCs are applicable seeding cells for SCI repair because of the maintained IL10 level in serum and infiltration into injured site.

Ideally, a single administration of MSCs is sufficient to exert its regenerative effect. However, the transplanted cells can survive for only a limited time in harsh environments. In many clinical studies, patients were treated with stem cells multiple times to obtain positive results [63–67]. For example, a Korean research team found that, despite the increase in the number of transplanted cells, the number of administrations of MSCs was reduced from three times in the preliminary study to one time in the phase III trial, resulting in poor treatment effect [68]. In addition, it is impractical to repeatedly carry out local administration of seeding cells to injured spinal cord in clinic. Therefore, we need to optimize the cell delivery method upon considering clinical application [62, 69]. Additional injections could provide more living cells that remodel the wound environment to promote regeneration [64]. In our study, we performed IL10-MSC therapy through intravenous administration every 2 weeks in an SCI model, mimicking repeated clinic application for patients with SCI in the future, leading to better therapeutic effects.

Microglia cells are innate immune cells of the CNS and play a crucial role in SCI recovery. Victor et al. showed that the absence of microglia destroyed the tissue of the astrocytic scar and reduced the number of neurons and oligodendrocytes at the injured site, leading to an impairment of functional recovery after SCI [70]. After SCI, microglia and macrophages infiltrated from the peripheral system are activated into two kinds of phenotypes, namely alternately activated M2 and classically activated M1. In general, M2 and M1 microglia/macrophages have anti-inflammatory and inflammatory roles, respectively [43, 70–72]. Anat Shemer et al. confirmed that IL10 played a critical role in preventing excessive activation of deleterious microglia in cognitive dysfunction of LPS-challenged IL10-receptor-deficient mice [73]. In the present study, the grafting of IL10-MSCs could markedly increase the proportion of M2 microglia (CD11b^hi^/CD45^lo^/CCR2^−^/Arg1^+^ cells) and infiltrated macrophages (CD11b^lo^/CD45^hi^/CCR2^+^/Arg1^+^ cells), meanwhile reducing M1 infiltrated macrophages (CD11b^lo^/CD45^hi^/CCR2^+^/iNOS^+^ cells). Most research on SCI repair has focused on local immune responses in injured spinal cord. However, there are few studies on the sequential variation of peripheral immune responses caused by SCI. In our previous study, we found that the spinal immune responses of SCI affected the changes of systemic immune responses, which in turn regulated the pathophysiological changes that occur in the injured area [37]. Because we performed IL10-MSC treatment through intravenous injection and found that serum IL10 could last 3 days after IL10-MSC transplantation (Additional file 1: Fig. S3), we wondered whether this treatment also affects the systemic immune responses. Analysis of specific subtypes of T cells in peripheral blood after SCI showed that the ratio of CD4^+^ FoxP3^+^ Treg to CD4^+^IL4^+^ Th2 subgroups in the IL10-MSCs-treated group significantly increased, while the proportion of the CD4^+^IFN-γ^+^ Th1 subgroup was significantly reduced (Additional file 1: Fig. S2), which was consistent with experimental data in vitro (Additional file 1: Fig. S1). Recent evidence has shown that IFN-γ-KO mice after contusive SCI had a significantly lower degree of impairment, indicating that the adaptive immune response biased toward CD4^+^IFN-γ^+^ TH1 was harmful to SCI recovery [74, 75]. On the other hand, Treg cells counteracted the inflammatory response by suppressing the inflammatory activity of innate and adaptive immune cells and by secreting factors involved in tissue repair [76, 77]. Treg cells secreted IL10 to induce Vav1 autocrine–paracrine signal transduction and activate Rac1 to promote apoptotic cell engulfment in macrophages. Therefore, enhancing Treg cells provides a novel strategy to alleviate the inflammatory responses in SCI [78]. Apart from preferential inhibition of Th1, our results showed that IL10-MSCs also significantly induced the differentiation of the Treg cell subset. Thus, we speculated that IL10-MSCs had the beneficial characteristics of T cells in peripheral blood and played an important role in the recovery of SCI.

On the basis of the strong central and peripheral immune regulations of IL10-MSCs, we further explored the potential protective mechanism by which IL10-MSC treatment achieved better therapeutic outcomes in SCI repair. It is well known that the restoration of residual neurons or formation of newborn neurons is crucial to recovery of locomotor functions after SCI [79, 80]. It was reported that the decrease in M1 activation of microglia/macrophages and the increase in M2 activation reduced their pro-inflammatory properties, thereby improving the prognosis after SCI [70]. Therefore, we speculated that the IL10-MSC-induced phenotypic switches of M2/M1 microglia/macrophages could regulate the regeneration of neurons and their axon growth. We observed that IL10-MSC treatment dramatically enhanced the regeneration of axons (NF-positive staining) and neurons (Tuj1-positive staining) in injured spinal cord, compared with MSC treatment. In vitro, IL10-MSC-derived conditioned medium could promote the switch of M1 to M2 phenotype in BV2 cells. In addition, we observed that the M1 microglia/macrophages induced by LPS + IFN-γ challenges decreased the survival and neuronal differentiation of NSCs, which was relieved by co-culture of IL10-MSCs. Immune cells in SCI repair are two-edged swords and have supportive or harmful effects on surrounding neurons depending on their activation state [81, 82]. Our results clearly indicated that IL10-MSC treatment inhibited the M1 subtype and preserved the M2 phenotype of microglia/macrophage at injured spinal cord, leading to an increase in neuronal regeneration and locomotion functional recovery.

**Fig. 7:**
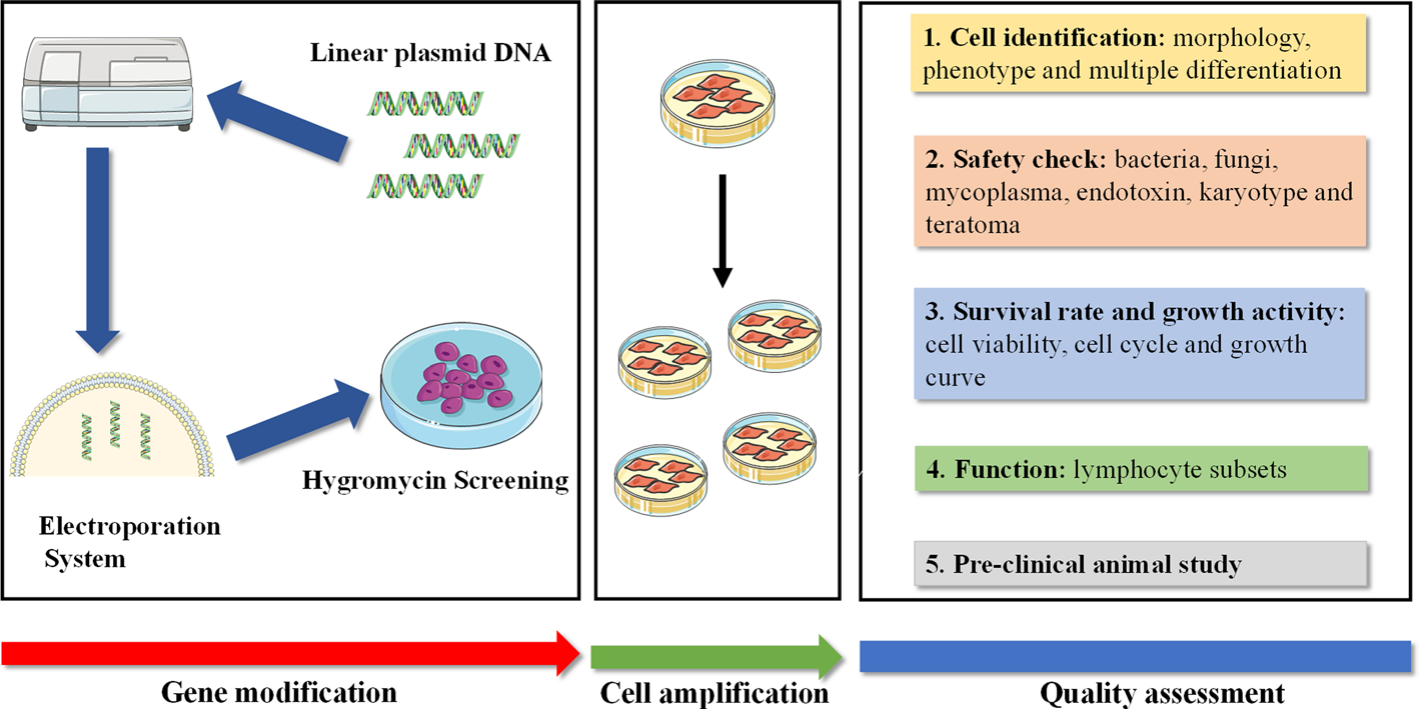
Schematic standard procedures of gene-modified MSCs. Firstly, the clinic-grade MSCs were isolated, identified, and amplified. The target gene was integrated into the vector and jointly introduced into MSCs, and then positive gene-modified MSCs were screened out. Secondly, the screened gene-modified MSCs were amplified to a sufficient amount. All the processes were performed with accordance to GMP guidelines. Thirdly, the systematic quality evaluations included but were not limited to surface marker profiles, general biological activity, safety, immune regulation, multiple lineage differentiation, tumorigenesis, and preclinical animal study

As a cell-therapeutic product, especially for gene-modified seeding cells, safety is a major concern when applied to recipients. In general, MSCs as a cell-based product should undergo extensive quality assessments to ensure they meet the requirement of guidelines and regulations of authorities. When an external gene is introduced into MSCs, it is probably randomly inserted into DNA sequences of chromosomes, which potentially decreases some biological activities or activates the signal pathways related to tumorigenesis, leading to an increased tumor risk to recipients when used in clinic. Thus gene-modified seeding cells should undergo more stringent cellular quality evaluation to meet the quality criteria of cell-therapeutic products. Although there are many studies regarding gene modification in cell-based therapy, they often ignored the systematic quality assessment of genetic modified cells as a cell-based product or performed only one fragment of quality assessment. To our knowledge, we are the first to perform a systemic quality evaluation for gene-modified clinic-grade MSCs to guarantee their safety and efficacy in clinic use. We propose a standard manufacture procedure regarding gene-modified MSCs as shown in Fig. 7.

## Conclusions

We established highly overexpressing IL10-MSCs in accordance with the criteria of cell-based products. After comprehensive quality evaluation, qualified IL10-MSCs were applied to SCI repair in a mouse model by multiple intravenous injections. IL10-MSC treatment achieved much better therapeutic outcomes, including reinforced locomotor improvement and neuronal regeneration, by promoting the switch of M2/M1 microglia/macrophages at injured sites and enhancing anti-inflammatory responses in the peripheral immune system. Our findings indicate that the strategy of IL10 genetically modified MSCs is applicable to SCI treatment, providing new insights into the benefits of systemic administration of cell-based therapeutic product.

## Supplementary Information

**Additional file 1: Figure S1:** The immunomodulatory effects of IL10-MSCs and MSCs. **A**, **B** IL10-MSCs or MSCs administration significantly inhibited the proliferation of CFSE-labeled PBMC via co-culturing. **C**, **D** MSCs treatment promoted the maturation of Treg subpopulation (CD4^+^CD25^+^FoxP3^+^) in PBMCs induced by IL-2. IL10-MSCs treatment significantly enhanced the Treg subset differentiation, compared with naïve MSCs group (***p < 0.001). **E**, **F** IL10-MSCs treatment could suppress the activation and differentiations of CD4^+^ T cells into Th1 subset (CD4^+^IFN-γ^+^). **G**, **H** IL10-MSCs or MSCs administration inhibited the differentiation of TH17 subpopulation (CD4^+^IL17A^+^), but without statistical difference between the two groups. **Figure S2:** Proportion of peripheral T cell subsets in SCI mice after cell grafting at 14 days. **A** Representative dot plots show the percentage of gated CD4+ T-cells compared to control. **B**, **C** The FACS analysis of Treg subset (CD4^+^CD25^+^FoxP3^+^). SCI resulted in a decrease in peripheral Treg subset, but the decrease could be rescued by IL10-MSCs treatment. **D**, **E** The FACS analysis of peripheral Th1 (CD4^+^IFN-γ^+^) subset. The proportion of Th1 subset was increased in SCI model group. Both IL10-MSCs and MSCs treatment could decrease the proportion of peripheral Th1 subset, and IL10-MSCs treatment further decreased the Th1 subpopulation, compared with the MSCs treatment (*p < 0.05). **F**, **G** The subpopulation of Th2 (CD4^+^IL4^+^) was markedly decreased in SCI model group. IL10-MSCs or MSCs treatment enhanced the differentiation of CD4^+^ T cells into Th2 subpopulation. The proportion of Th2 subset in IL10-MSCs group was significantly higher than that in MSCs group (*p < 0.05). **Figure S3:** **A**, **B** IL10-MSCs treatment did not alter the ratio of resident microglia (CCR2^−^/CD11b^hi^CD45^lo^) and infiltrating macrophages (CCR2^+^/CD11b^lo^CD45^hi^). **C** The serum IL10 level was detected using ELISA method at indicated time points after IL10-MSCs transplantation. We successfully detected the IL10 in serum at day 3 post transplantation. **D** The transplanted GFP labeled IL10-MSCs were traced in injured spinal cord by immunostaining. The many GFP-positive cells were observed in GFP labeled IL10-MSCs treatment, but negative in unlabeled IL10-MSCs treatment in injured site. Scale bars = 100 μm.

## Data Availability

All data generated or analyzed during this study are included in this published article. The datasets used and/or analyzed during the current study are available from the corresponding author on reasonable request.

## Abbreviations

hUCMSCs: Human umbilical-cord-derived mesenchymal stem cells
SCI: Spinal cord injury
NSCs: Neural stem cells
H&E: Hematoxylin–eosin
ELISA: Enzyme-linked immunosorbent assay
GFAP: Glial fibrillary acidic protein
Tuj1: βIII-tubulin
NF: Neurofilament
