# Supplementary material for: Interleukin-10 genetically modified clinical-grade mesenchymal stromal cells markedly reinforced functional recovery after spinal cord injury via directing alternative activation of macrophages
Source: Cell Mol Biol Lett. 2022 Mar 17;27:27. doi: 10.1186/s11658-022-00325-9 (PMC8931978; doi:10.1186/s11658-022-00325-9)
Supplement: Supplementary file 1 — Additional file 1: Figure S1: The immunomodulatory effects of IL10-MSCs and MSCs. A, B IL10-MSCs or MSCs administration significantly inhibited the proliferation of CFSE-labeled PBMC via co-culturing. C, D MSCs treatment promoted the maturation of Treg subpopulation (CD4+CD25+FoxP3+) in PBMCs induced by IL-2. IL10-MSCs treatment significantly enhanced the Treg subset differentiation, compared with naïve MSCs group (***p < 0.001). E, F IL10-MSCs treatment could suppress the activation and differentiations of CD4+ T cells into Th1 subset (CD4+IFN-γ+). G, H IL10-MSCs or MSCs administration inhibited the differentiation of TH17 subpopulation (CD4+IL17A+), but without statistical difference between the two groups. Figure S2: Proportion of peripheral T cell subsets in SCI mice after cell grafting at 14 days. A Representative dot plots show the percentage of gated CD4+ T-cells compared to control. B, C The FACS analysis of Treg subset (CD4+CD25+FoxP3+). SCI resulted in a decrease in peripheral Treg subset, but the decrease could be rescued by IL10-MSCs treatment. D, E The FACS analysis of peripheral Th1 (CD4+IFN-γ+) subset. The proportion of Th1 subset was increased in SCI model group. Both IL10-MSCs and MSCs treatment could decrease the proportion of peripheral Th1 subset, and IL10-MSCs treatment further decreased the Th1 subpopulation, compared with the MSCs treatment (*p < 0.05). F, G The subpopulation of Th2 (CD4+IL4+) was markedly decreased in SCI model group. IL10-MSCs or MSCs treatment enhanced the differentiation of CD4+ T cells into Th2 subpopulation. The proportion of Th2 subset in IL10-MSCs group was significantly higher than that in MSCs group (*p < 0.05). Figure S3: A, B IL10-MSCs treatment did not alter the ratio of resident microglia (CCR2−/CD11bhiCD45lo) and infiltrating macrophages (CCR2+/CD11bloCD45hi). C The serum IL10 level was detected using ELISA method at indicated time points after IL10-MSCs transplantation. We successfully detected the IL10 in seru [file 11658_2022_325_MOESM1_ESM.docx]

**Additional Materials**

**Materials and methods**

**Immunomodulation assays of IL10-MSCs**

The immunomodulatory function of MSCs is the basis for treating various diseases such as SCI and osteoarthritis, which is recommended by ISCT as the efficacy release standard for advanced clinical trials [1]. We wondered whether the immunomodulation function of L10-MSCs was altered by IL10 gene modification. IL10-MSCs were co-cultured with human peripheral blood mononuclear cells (PBMCs) to determine their differentiation effects on Th1, Th17 and regulatory T cells (Tregs) as briefly described as follows. IL10-MSCs or naïve MSCs were inoculated into 6-well plates (1 × 10^5^/well), and incubated with Roswell Park Memorial Institute (RMPI) 1640 medium complemented with 10% FBS for 24 hours. The human PBMCs were prepared from human peripheral blood by density gradient centrifugation with a Ficoll Hypaque (AXISSHIELD, NOR). Then PBMCs were inoculated into 6-well plates in the presence or absence of IL10-MSCs (IL10-MSCs/PBMCs ratio, 1:10). For Treg population determination, rhIL-2 (5 ng/mL) was added to the medium. After 3 days of co-cultivation, T cells were collected and stimulated with 500 μL of 20 mM phytohemagglutinin-M (PHA-M, 10576015, Gibco) for 5 h. Cells were first incubated with CD3-Percp and CD8-APC antibody solutions (BD, USA) at room temperature for 15 minutes. Then, cells were fixed and permeabilized using the cell fixation and permeabilization kit (FMS, China) according to the manufacturer's instructions. After washing with 1× PBS, the intracellular IFN-γ and IL-17A were detected by incubating with IFN-γ-FITC and IL-17A-PE antibodies. To detect Treg subpopulation, non-adherent PBMCs were collected and assayed according to the manufacturer's instructions (eBioscience, USA). Cells were analyzed by flow cytometry (BD FACSAria™, USA), and data were analyzed by FACS software (Additional Figure S1).

**Assaying peripheral T-cells subsets after IL10-MSCs treatment in SCI mice**

At day 14 after SCI, mice were anesthetized with 1.25% tribromoethanol, and then the peripheral bloods were collected via retro-orbital plexus. A total of 50 μl peripheral blood were suspended in individual tubes and cultured in RPMI 1640 medium (Gibco, 12633012, Life Technologies Co., USA), supplemented with 10% FBS (10099141, Life Technologies Co.), 100 units/mL of penicillin and 100 µg/mL of streptomycin (10378016, Life Technologies Co.,), and 2.5 µL T-cell induction Cocktail (004975-93; eBioscience, USA) in a 5% CO2 incubator at 37 °C for 5 hours.

The following fluorochrome-labeled antibodies were used for flow cytometric analysis, according to the manufacturers’ protocols: anti-mouse CD4-FITC(11-0041-82, eBioscience), anti-mouse IFN-γ-APC, 17-7311-82, eBioscience), anti-mouse IL-17A-PE(12-7177-81, eBioscience), anti-mouse CD25-APC (17-0251-82, eBioscience), anti-mouse FoxP3-PE (12-5773-82; eBioscience), and anti-mouse IL-4-PE-Cy7 (25-7042-42, eBioscience) antibodies. Finally, the stained cells were analyzed by a FACSCantoTM flow cytometer (BD Biosciences) and analyzed using FlowJo V10 software (BD Biosciences) (Additional Figure S2).

**Tracing transplanted IL10-MSCs post-SCI**

In order to determine whether transplanted IL10-MSCs could home to the injured sites via tail vein injection, IL10-MSCs were labeled by GFP and injected via tail vein after SCI. After day 3, the mice were sacrificed and sectioned the injured spinal cord. The immunofluorescence was performed using anti-GFP antibody to detect whether the transplanted GFP-labeled IL10-MSCs could infiltrate into the injured site (Additional Figure S3).


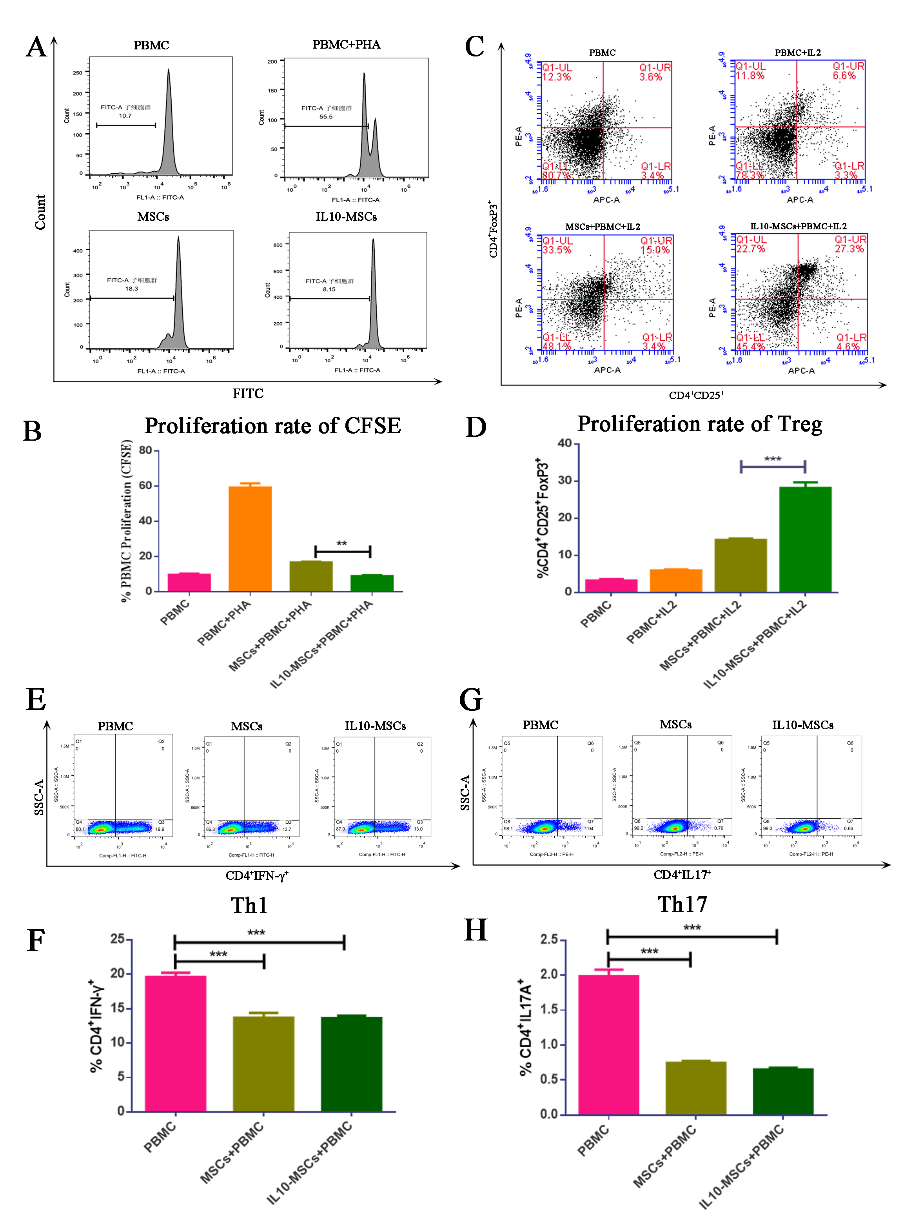


**Additional Figure S1:** The immunomodulatory effects of IL10-MSCs and MSCs. (A and B) IL10-MSCs or MSCs administration significantly inhibited the proliferation of CFSE-labeled PBMC via co-culturing. (C and D) MSCs treatment promoted the maturation of Treg subpopulation (CD4^+^CD25^+^FoxP3^+^) in PBMCs induced by IL-2. IL10-MSCs treatment significantly enhanced the Treg subset differentiation, compared with naïve MSCs group (****p* < 0.001). (E and F) IL10-MSCs treatment could suppress the activation and differentiations of CD4^+^ T cells into Th1 subset (CD4^+^IFN-γ^+^). (G and H) IL10-MSCs or MSCs administration inhibited the differentiation of TH17 subpopulation (CD4^+^IL17A^+^), but without statistical difference between the two groups.


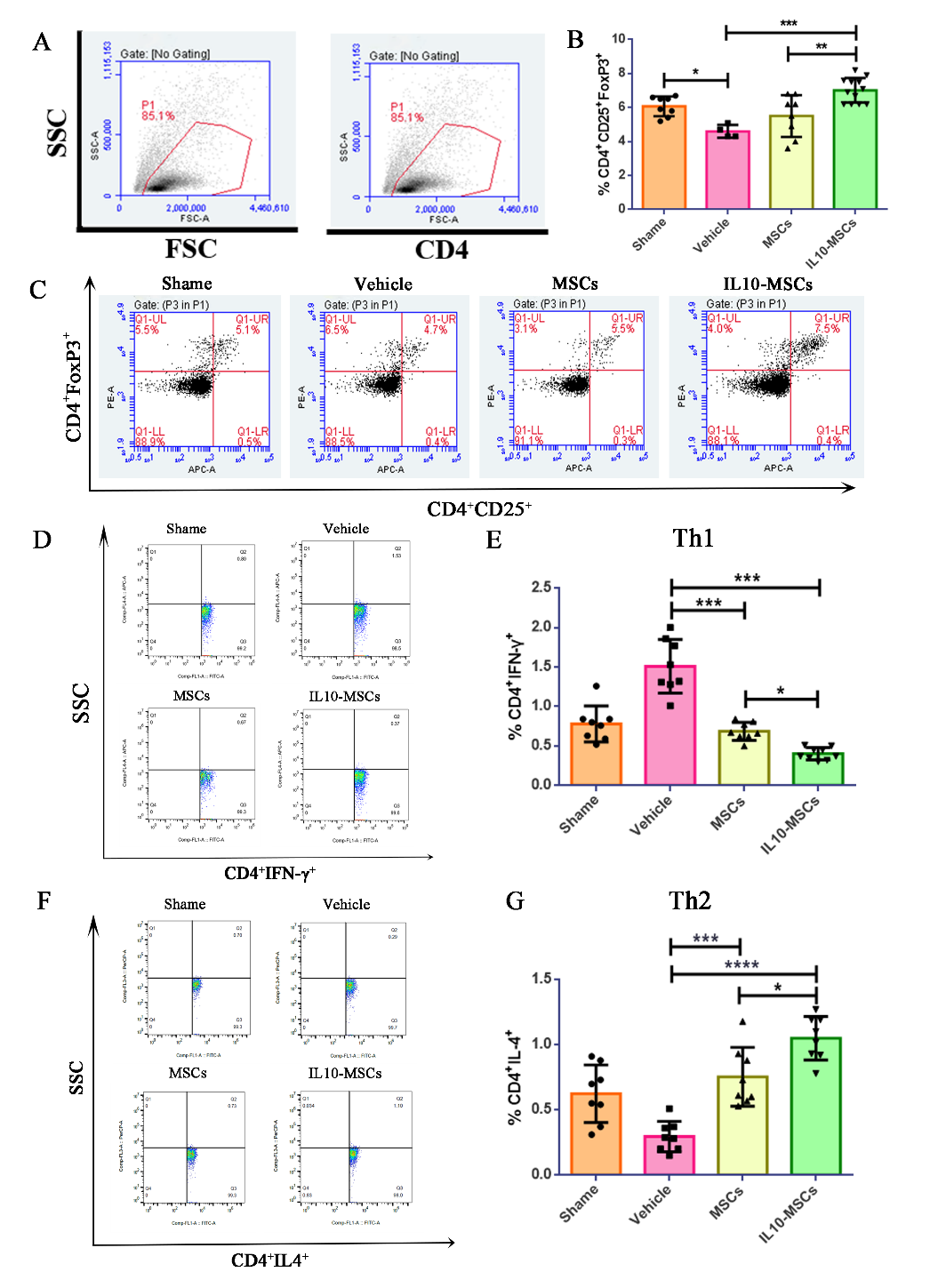


**Additional Figure S2:** Proportion of peripheral T cell subsets in SCI mice after cell grafting at 14 days. (A) Representative dot plots show the percentage of gated CD4+ T-cells compared to control. (B-C) The FACS analysis of Treg subset (CD4^+^CD25^+^FoxP3^+^). SCI resulted in a decrease in peripheral Treg subset, but the decrease could be rescued by IL10-MSCs treatment. (D-E) The FACS analysis of peripheral Th1 (CD4^+^IFN-γ^+^) subset. The proportion of Th1 subset was increased in SCI model group. Both IL10-MSCs and MSCs treatment could decrease the proportion of peripheral Th1 subset, and IL10-MSCs treatment further decreased the Th1 subpopulation, compared with the MSCs treatment (**p* < 0.05). (F-G) The subpopulation of Th2 (CD4^+^IL4^+^) was markedly decreased in SCI model group. IL10-MSCs or MSCs treatment enhanced the differentiation of CD4^+^ T cells into Th2 subpopulation. The proportion of Th2 subset in IL10-MSCs group was significantly higher than that in MSCs group (**p* < 0.05).


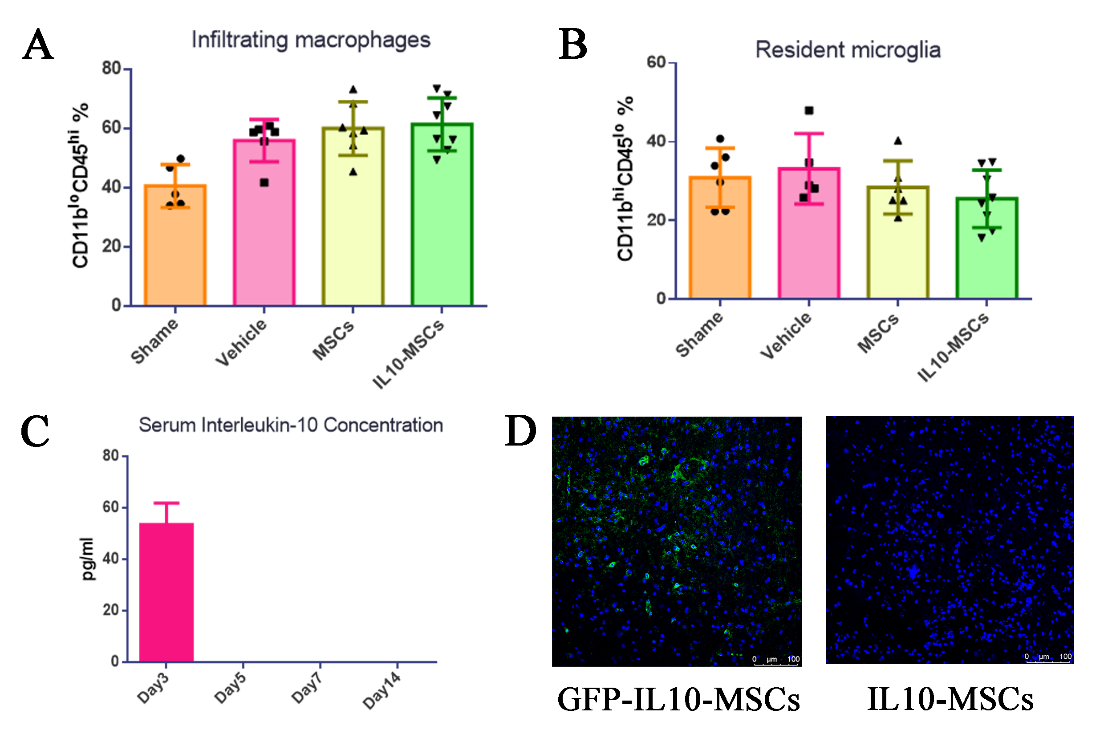


**Additional Figure S3:** (A-B) IL10-MSCs treatment did not alter the ratio of resident microglia (CCR2^-^/CD11b^hi^CD45^lo^) and infiltrating macrophages (CCR2^+^/CD11b^lo^CD45^hi^). (C) The serum IL10 level was detected using ELISA method at indicated time points after IL10-MSCs transplantation. We successfully detected the IL10 in serum at day 3 post transplantation. (D) The transplanted GFP labeled IL10-MSCs were traced in injured spinal cord by immunostaining. The many GFP-positive cells were observed in GFP labeled IL10-MSCs treatment, but negative in unlabeled IL10-MSCs treatment in injured site. Scale bars = 100 μm.

**Reference**

1. Cao Y, Sun H, Zhu H, Zhu X, Tang X, Yan G, Wang J, Bai D, Wang J, Wang L, et al: **Allogeneic cell therapy using umbilical cord MSCs on collagen scaffolds for patients with recurrent uterine adhesion: a phase I clinical trial.** *Stem Cell Res Ther* 2018, **9:**192.
